# A Novel Model for Papillomavirus-Mediated Anal Disease and Cancer Using the Mouse Papillomavirus

**DOI:** 10.1128/mBio.01611-21

**Published:** 2021-07-20

**Authors:** Simon Blaine-Sauer, Myeong-Kyun Shin, Kristina A. Matkowskyj, Ella Ward-Shaw, Paul F. Lambert

**Affiliations:** a McArdle Laboratory for Cancer Research, University of Wisconsin School of Medicine and Public Health, Madison, Wisconsin, USA; b Department of Pathology and Laboratory Medicine, University of Wisconsin School of Medicine and Public Health, Madison, Wisconsin, USA; c University of Wisconsin Carbone Cancer Center, Madison, Wisconsin, USA; d William S. Middleton VA Medical Center, Madison, Wisconsin, USA; University of North Carolina, Chapel Hill

**Keywords:** DMBA, HPV, anal cancer, infection, mouse, papillomavirus, preclinical model, transition zone, MmuPV1

## Abstract

Up to 95% of all anal cancers are associated with infection by human papillomavirus (HPV); however, no established preclinical model exists for high-grade anal disease and cancer mediated by a natural papillomavirus infection. To establish an infection-mediated model, we infected both immunocompromised NSG and immunocompetent FVB/NJ mice with the recently discovered murine papillomavirus MmuPV1, with and without the additional cofactors of UV B radiation (UVB) and/or the chemical carcinogen 7,12-dimethylbenz(a)anthracene (DMBA). Infections were tracked via lavages and swabs for MmuPV1 DNA, and pathology was assessed at the endpoint. Tissues were analyzed for biomarkers of viral infection and papillomavirus-mediated disease, and the localization of viral infection was investigated using biomarkers to characterize the anal microanatomical zones.

## INTRODUCTION

An estimated 9,090 people in the United States will be diagnosed with anal cancer in 2021, representing approximately 0.5% of all new cancer diagnoses (1). Despite the availability of prophylactic human papillomavirus (HPV) vaccines, the incidence and mortality of anal cancers has consistently risen over the past several decades (2, 3). Furthermore, current standard-of-care treatments for anal cancer can have significant adverse effects on patients' quality of life, including anal dysfunction, characterized by increased diarrhea and incontinence, impaired social functioning, and sexual dysfunction (4–6). The vast majority of anal squamous cell carcinomas are associated with HPV infection. Studies have found HPV positivity rates in anal carcinomas ranging from 84% to 95%, with the high-risk mucosotropic strain HPV16 being associated with between 73% and 89% of all malignancies (7–10). Additional risk factors for the development of anal carcinoma include infection with HIV, immunosuppression following organ transplantation, smoking, a higher lifetime number of sexual partners, and a history of receptive anal intercourse (7, 11–13). Men who have sex with men (MSM) are at particular risk for developing anal cancer compared to the general population, although overall incidence of anal cancer is higher in women than in men and a history of neoplastic disease in the female reproductive tract is an additional risk factor for the development of anal disease (1, 13–16).

Papillomaviruses are highly species specific, and the lack of a papillomavirus that infects the common laboratory mouse limited the study of papillomavirus-mediated disease in animal models prior to the discovery of a mouse papillomavirus, MmuPV1, in 2010 (17). In lieu of a tractable infection-driven model, transgenic mouse models expressing the high-risk HPV16 oncogenes E6 and/or E7 have been highly valuable in the study of papillomaviruses, especially in elucidating the biochemical mechanisms of HPV-mediated carcinogenesis (18, 19). We previously developed a mouse model of anal cancer using HPV16 transgenic mice in combination with the chemical carcinogen 7,12-dimethylbenz(a)anthracene (DMBA). This model has proven useful to us and colleagues in the HPV field in understanding the contributions of viral and host oncogenes in anal carcinogenesis, the role of autophagy in anal cancer, and as a preclinical model for testing therapeutic drugs (20–27). However, there are limitations to this transgenic model of anal cancer. In disease and cancers arising from a natural infection event, the infected cells must compete with uninfected neighboring cells for growth and survival. This depends upon expression of virally encoded proteins that are necessary to maintain the presence of the viral genome, drive continued expression of viral genes, and evade host immune responses. Natural papillomavirus infections also likely involve an ability of the virus to infect specific subtypes of cells within a given tissue. Transgenic mouse models do not recapitulate most of these facets of the natural disease. Thus, development of a model for anal disease and cancer mediated by a natural infection is warranted.

MmuPV1 is a Pi-family papillomavirus that infects the common laboratory mouse Mus musculus at both cutaneous and mucosal sites and has oncogenic potential in the skin, cervicovaginal tract, and head and neck, despite differences in the molecular activities of its viral E6 and E7 proteins from the high-risk alpha-HPVs (28–37). The Christensen laboratory previously reported that the anus of immunocompromised *FoxN1^nu/nu^* and NSG mice is susceptible to infection with MmuPV1, particularly in the area around the anal transformation zone, and that MmuPV1 can mediate low-grade dysplasia in *FoxN1^nu/nu^* mice (32, 38–40). They also reported that MmuPV1 is able to persist in the anal tract of *FoxN1^nu/+^* heterozygotes, although virus was not detected by histopathological analysis in these mice at the endpoint (32). In fully immunocompetent C57BL/6J mice, they were unable to detect infection in the anal tract by 6 weeks postinfection, even after partial immunosuppression with CD4 and CD8 antibodies. We sought to investigate whether MmuPV1 could induce high-grade disease and cancer of the anus in immunodeficient mice and whether MmuPV1 is able to persist and induce neoplastic anal disease in the fully immunocompetent FVB/NJ strain, which we have previously found is susceptible to MmuPV1-associated cancers at other sites (33–35, 41).

Here, we present a model of anal disease and cancer mediated by infection with MmuPV1. We report for the first time that MmuPV1 infection is sufficient to efficiently induce high-grade squamous intraepithelial lesions (HSIL) in NSG immunocompromised mice by 4.5 months postinfection. We further demonstrate that MmuPV1 exhibits carcinogenic potential in NSG mice when the infected region is exposed to the chemical carcinogen DMBA. In the FVB/NJ strain, we demonstrate that the virus is able to persist for up to 6 months in immunocompetent mice irradiated with UV B radiation (UVB) and that viral infection in combination with DMBA treatment mediates anal HSIL and contributes to the formation of anal cancers. We also examine the role of the anal microanatomy in MmuPV1 infection and disease, demonstrating that the virus preferentially gives rise to lesions in the anal transition zone independent of infection method. This model opens up new possibilities for studying the role of papillomavirus infection in anal cancers and precancerous disease and provides a valuable new preclinical model for testing the efficacy of potential therapies against HPV-mediated anal neoplasia and cancer.

## RESULTS

### MmuPV1 infection persists and mediates high-grade squamous intraepithelial lesions in the anal tract of immunocompromised NSG mice.

To develop a model of papillomavirus-mediated disease in the anal canal, we first infected NOD scid gamma (NSG) mice with MmuPV1. NSG mice are immunocompromised, being unable to mount functional B, T, or NK cell responses and having impaired cytokine function (42). Infections were carried out using a Greer pick (Greer Laboratories, Inc., Lenoir, NC) allergen skin-testing device. The use of this pick for infecting mice with MmuPV1 has been previously described by our group in the context of the oral cavity and oropharynx (35, 37) and allows us to simultaneously wound the epithelium and expose the underlying basal layer of the epithelium to virus, as it is thought that infections must initiate through infection of basal epithelial cells (43). Infection was performed on both the dorsal and ventral sides of the anal canal.

**FIG 1:**
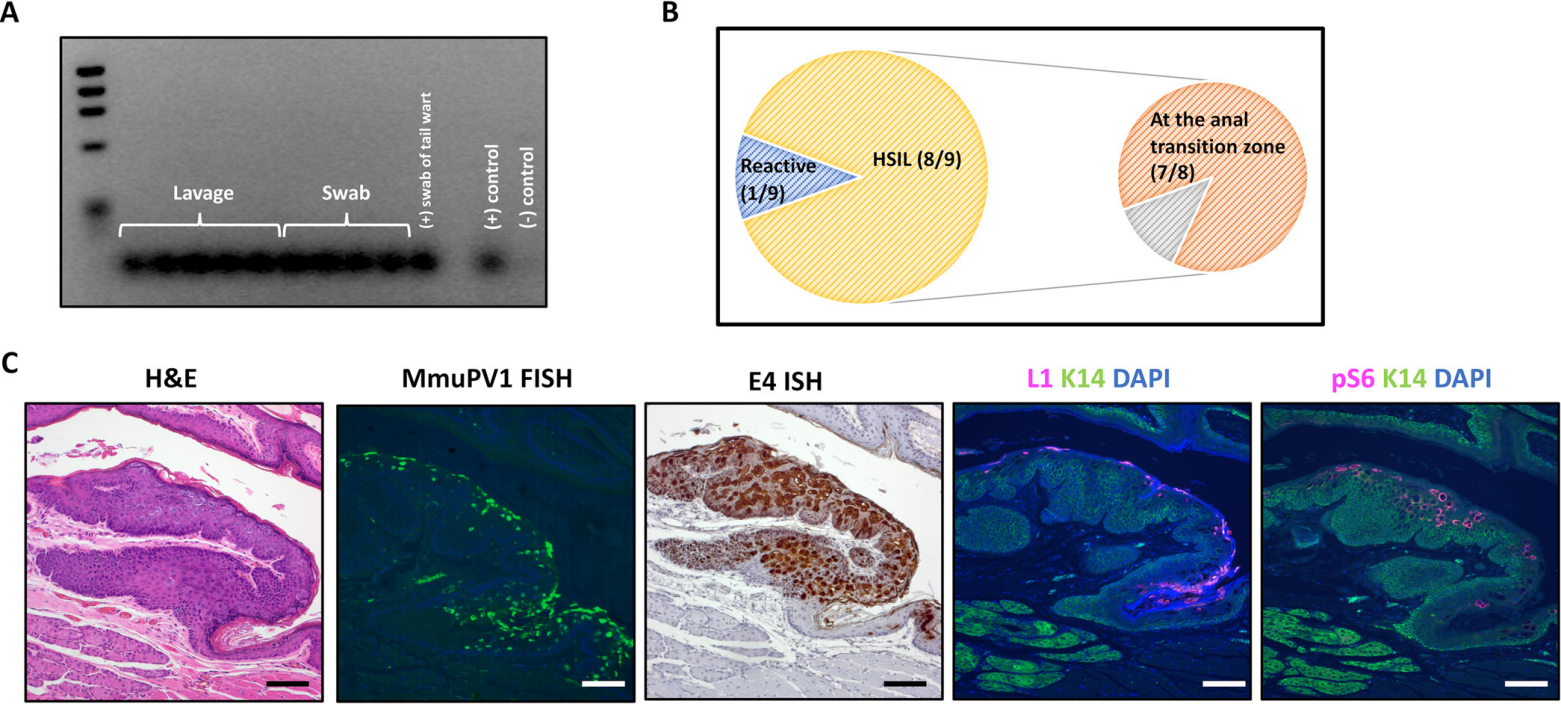
MmuPV1 persists and mediates high-grade disease in the anal tract of NSG mice by 20 weeks postinfection. (A) MmuPV1 was detected in the anal tracts of nine of nine (100%) mice by anal lavage or swab, followed by PCR for MmuPV1 DNA at the 20-week postinfection study endpoint. (B) Incidence and localization of anal disease at the endpoint. A perianal gland adenoma was also noted in one mouse with high-grade dysplasia. (C) Virus was detected within lesions by FISH for MmuPV1 DNA, ISH to the E4 gene using RNAScope, and immunofluorescence for the L1 capsid protein. Phospho-S6, an established biomarker for papillomavirus-mediated disease, was also highly expressed. All scale bars equal 100 μm.

Mice were monitored over the course of the 20-week study. Of the nine mice, only one developed a clear overt exophytic lesion by the endpoint, although several displayed roughness and discoloration in the anal mucosa and around the edges of the anal tract, indicative of possible disease. To determine the persistence of the virus, anal tracts of the mice were swabbed with a cotton-tipped pick or were lavaged with phosphate-buffered saline (PBS) at the endpoint using a technique adapted from Hu and colleagues (39), and PCR was run with primers specific to the MmuPV1 genome. Nine of nine mice were positive for viral DNA in the anus at the 20-week endpoint, demonstrating 100% infection efficiency and persistence (Fig. 1A). Hematoxylin and eosin (H&E)-stained slides were scored by a trained gastrointestinal pathologist (K.A.M.) for disease. At the 20-week endpoint, eight of nine mice (89%) developed high-grade squamous intraepithelial lesions (HSIL), including the mouse with the overt exophytic lesion, while one mouse was scored as reactive (Fig. 1B). Disease was localized to the anal transition zone in seven of the eight (87.5%) mice with HSIL. The anal transition zone, also referred to as the squamocolumnar junction or the anorectal junction, is the microanatomical area where the epithelium transitions from the columnar epithelium of the rectum to the stratified squamous epithelium that comprises the most caudal region of the anal canal and the perianal skin (44, 45) and had been previously shown by Cladel and colleagues to be susceptible to MmuPV1 infection (40).

Fluorescent *in situ* hybridization (FISH) for MmuPV1 DNA, RNAScope hybridization for viral E4-containing transcripts, and immunofluorescence for the viral L1 capsid protein were performed to detect the presence and location of viral infection in the anal tract tissues at the endpoint. Results for a representative lesion are shown (Fig. 1C). Strong detection of viral DNA, mRNA, and capsid protein at sites of disease indicate a robust and productive MmuPV1 infection at the 20-week endpoint. Regions of persistent infection and disease also had high levels of phospho-S6, a protein that is phosphorylated during PIK3/mTOR signaling. Phopho-S6 is an established biomarker for papillomavirus-mediated disease and has been previously used by our group as a biomarker for anal disease arising in HPV16 transgenic mice (22, 23, 46).

### MmuPV1 exhibits carcinogenic potential in NSG mice in combination with the chemical carcinogen DMBA.

**FIG 2:**
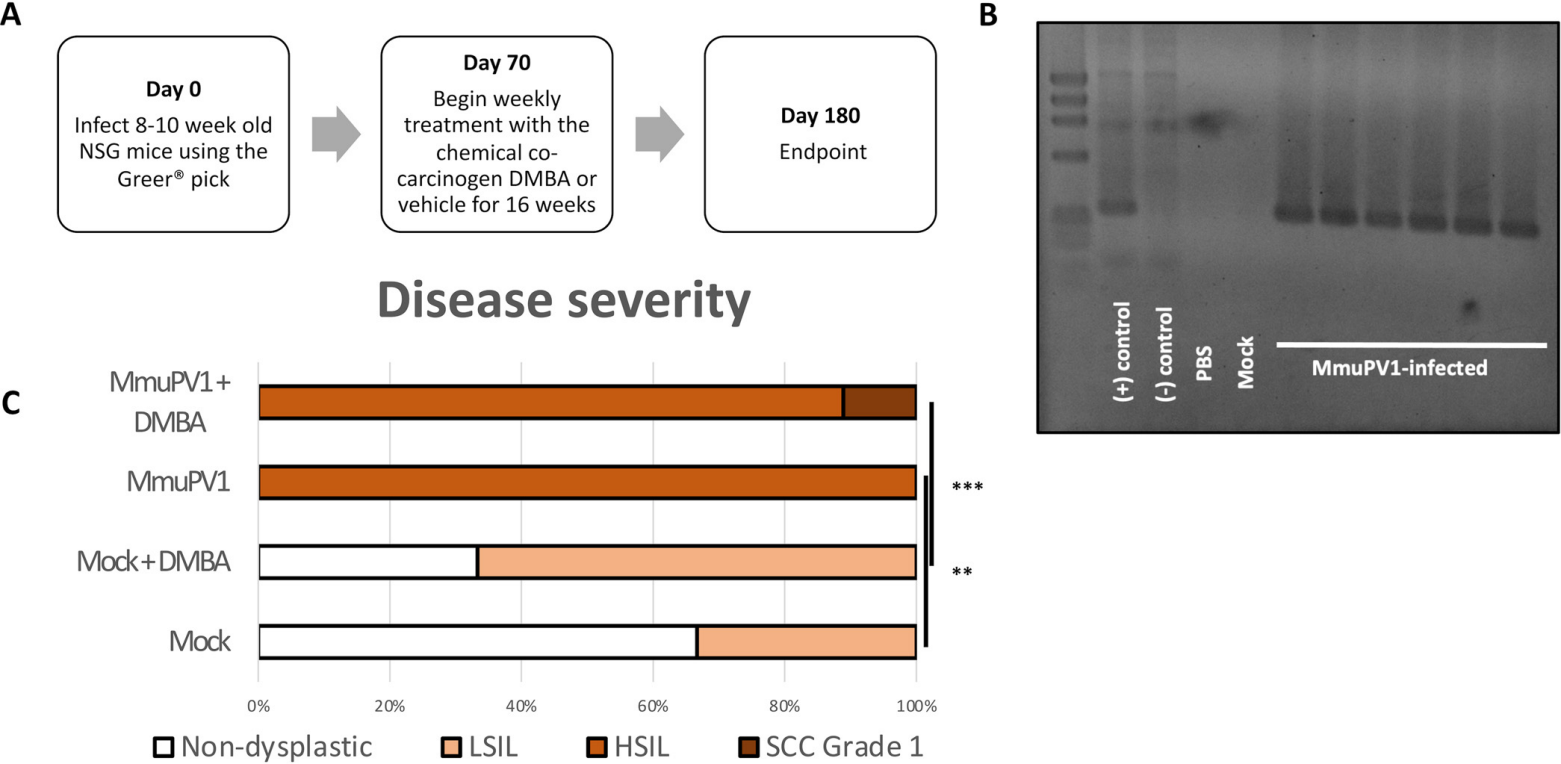
MmuPV1 in combination with the chemical carcinogen DMBA has carcinogenic potential in NSG mice. (A) Outline of experimental design. (B) Lavage of a representative subset of infected NSG mice at 7 weeks postinfection, demonstrating 100% infection efficiency. (C) Blinded scoring of disease severity in mice performed by a trained gastrointestinal pathologist. **, *P* < 0.01; ***, *P* < 0.001.

We next infected NSG mice as described above and, at 10 weeks postinfection, began topical treatment once weekly with 0.12 μmol the chemical carcinogen DMBA or the vehicle for 16 weeks (Fig. 2A). Our group has previously used DMBA as a cocarcinogen in our HPV16 transgenic model of anal carcinogenesis (22, 24). Anal lavage was performed on a subset of the mice at 7 weeks postinfection. MmuPV1 was detected by PCR in 100% of infected mice (Fig. 2B), consistent with results of our prior experiment (Fig. 1A).

**FIG 3:**
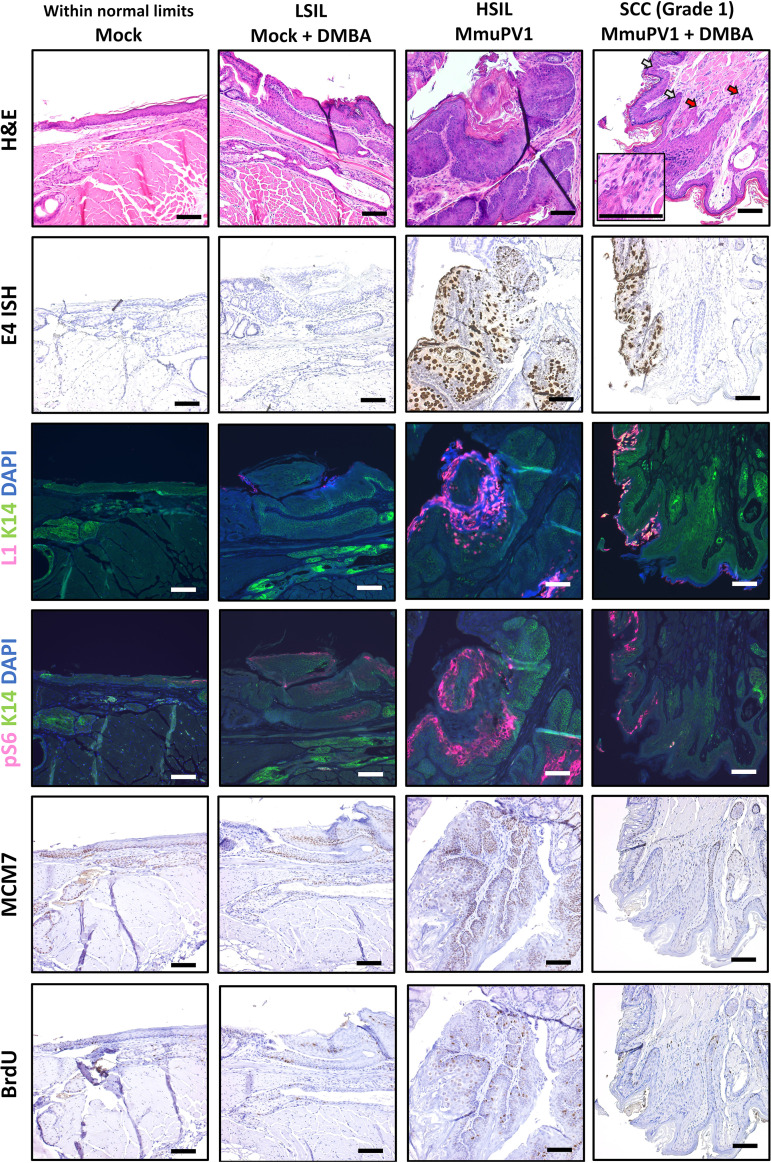
Biomarker analysis of MmuPV1-infected NSG mice. Representative tissues were stained for markers of viral infection (E4 RNAScope ISH and L1/K14 IF). Based on H&E staining and E4 RNAScope, a representative tissue for each group was further examined for established biomarkers of papillomavirus-mediated disease (pS6/K14 IF, MCM7 IHC) and proliferation (BrdU IHC). All images show tissues stained in the same experiment and were taken with constant exposure for biomarkers of interest. Red arrows within the H&E image mark areas of invasive carcinoma, and white arrows mark areas of dysplasia. The H&E inset shows a high-magnification image of a focal invasive region. All scale bars equal 100 μm.

We found that MmuPV1 infection alone induced HSIL in 100% (6/6) of MmuPV1-infected mice by 6 months postinfection (*P* = 0.0087, MmuPV1 only versus mock only). When combined with weekly DMBA treatment, one of nine (11%) mice progressed to invasive anal squamous cell carcinoma, grade 1, while the remaining mice were scored as HSIL (Table 1, Fig. 2C). Among mock-infected mice treated with DMBA, four of six (67%) progressed to LSIL but none progressed to high-grade disease or cancer (*P* = 0.00061, MmuPV1+DMBA versus mock+DMBA). MmuPV1 E4 DNA and RNA were detected with RNAScope throughout the high-grade lesions, and lesions were also positive for the L1 capsid protein, indicating a productive infection (Fig. 3). Notably, L1 expression characteristic of a productive viral infection is often lost in human anal disease as it progresses to squamous cell carcinoma, although L1 is still detected in some HSIL (47), and in cervical disease L1 expression is detected less frequently in HSIL than LSIL (48–51). In the sample containing the invasive anal squamous cell carcinoma, the strong MmuPV1 E4 RNAScope signal resided selectively in the adjoining dysplastic region and included nuclear signal that we have previously ascribed to MmuPV1 DNA, not RNA (52). In the area characterized histopathologically as invasive cancer (see red arrows in Fig. 3) nuclear DNA signal was absent, suggesting that viral DNA amplification, a hallmark of the productive phase of the papillomaviral life cycle, is lost as the cells invade. Trace cytoplasmic signal was detected within the invasive region by E4 and E6/E7 RNAScope ISH, but the level compared to background was not sufficiently high to be confidently characterized as true MmuPV1 RNA signal (see Fig. S1 in the supplemental material).

**TABLE 1:** Disease incidence in NSG mice, with and without DMBA treatment

Group	Total no. of mice	Sex^*b*^	Nondysplastic (*n*)	LSIL (*n*)	HSIL (*n*)	SCC grade 1 (*n*)
Mock	3	2 M, 1 F	2	1		
Mock + DMBA	6	4 M, 2 F	2	4		
MmuPV1 only^*a*^	6	2 M, 4 F			6	
MmuPV1 + DMBA^*a*^	9	5 M, 4 F			8	1

a There were no statistically significant sex-based differences in disease outcome in infected mice.

b M, male; F, female.

10.1128/mBio.01611-21.1 FIG S1 Viral signal was not reliably detected within the invasive NSG cancer by E4 or E6/E7 RNAScope. Nuclei were absent for ISH signal, indicating a lack of viral amplification. Trace cytoplasmic signal was detected near the invasive area, but it was not sufficiently above background elsewhere in the tissue to be confidently classified as true signal. All scale bars equal 100 μm. Download FIG S1, PDF file, 0.9 MB. Copyright © 2021 Blaine-Sauer et al. 2021 Blaine-Sauer et al. https://creativecommons.org/licenses/by/4.0/ This content is distributed under the terms of the Creative Commons Attribution 4.0 International license.

A representative lesion for each group was chosen based on H&E and RNAScope staining and analyzed for phosph-S6 as well as MCM7 and bromodeoxyuridine (BrdU) (mice injected with BrdU 1 h prior to sacrifice). MCM7 is an E2F-responsive gene that our group has previously shown is upregulated in papillomavirus-mediated disease, whereas BrdU is a nucleoside analog that is incorporated into DNA during active replication and is a marker for general proliferation (35, 53). BrdU was detected throughout the full thickness of the epithelium of the high-grade lesion, consistent with proliferation beyond the basal layer. In the squamous cell carcinoma, BrdU incorporation was also observed but at a much lower level, mostly at the invasive front of the anal squamous cell carcinoma (Fig. 3). Both pS6 and MCM7 were highly upregulated within areas of papillomavirus infection and disease compared to mock-infected tissue, with the pS6 protein most abundant in the upper epithelial layers and MCM7 expressed throughout the full thickness of the lesion. Neither MCM7 nor pS6 was as highly upregulated within the area of anal squamous cell carcinoma (see arrows in Fig. 3), as observed in the HSIL lesion.

### MmuPV1 is able to infect and persist in a subset of immunocompetent FVB/NJ mice irradiated with UVB.

**FIG 4:**
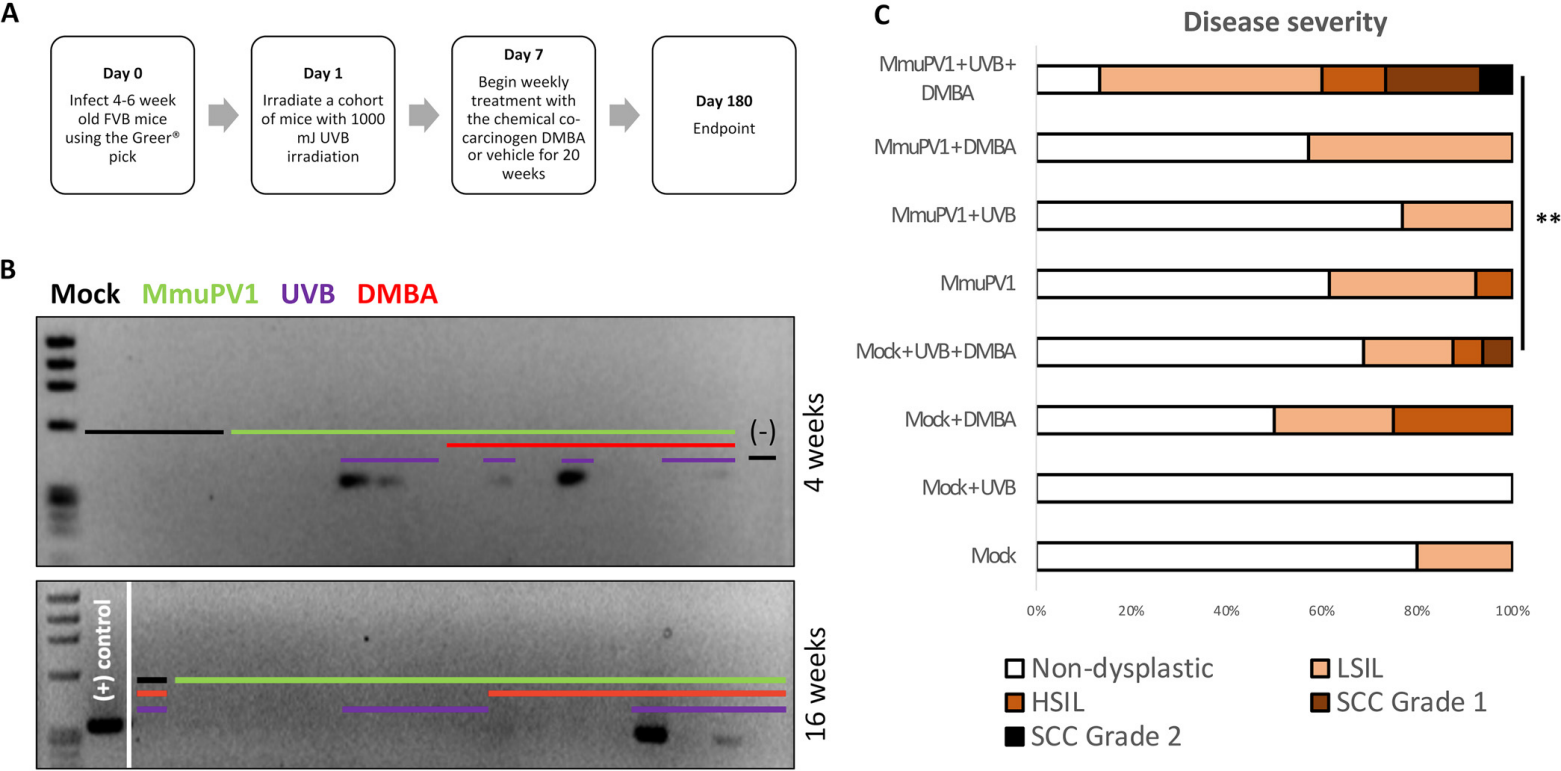
Mouse papillomavirus infects the anus of FVB/NJ immunocompetent mice and, together with UVB and DMBA, causes high-grade disease and cancer. (A) Outline of experimental design. (B) PCR analysis for MmuPV1 of lavages (4 weeks postinfection) and swabs (16 weeks postinfection) of a representative subset of FVB/NJ mice. (C) Disease severity of FVB/NJ mice infected with MmuPV1 with or without UVB irradiation and with or without DMBA, scored blindly by a trained gastrointestinal pathologist. **, *P* < 0.01.

We next infected FVB/NJ mice in order to establish a model of papillomavirus-mediated anal infection and disease in immunocompetent mice. FVB/NJ mice have previously been shown to be susceptible to persistent MmuPV1 infection in the skin, penis, cervicovaginal mucosa, and oral tract (30, 33–35, 41). Mice were infected using a Greer pick on the dorsal side of the anal tract. One day postinfection, a subset of mice from the infected and mock-infected groups were irradiated with 1,000 mJ/cm^2^ UVB spectral radiation. We have previously shown that UVB induces partial immune suppression in immunocompetent mice, sensitizing them to infection with MmuPV1 (34). Mice were then treated weekly with a topical administration of the chemical carcinogen DMBA or a vehicle-only solution for 20 weeks (Fig. 4A). Anal lavage was performed on a representative subset of mice at 4 weeks postinfection. Among infected mice treated with UVB irradiation, MmuPV1 was detected in five of seven samples (71%) (Fig. 4B). Notably, MmuPV1 was not detected at the 4-week time point in any mice that did not receive UVB irradiation, indicating that UVB-induced immunosuppression contributes to persistent MmuPV1 infection in the anal tract in FVB/NJ immunocompetent mice. To monitor viral persistence over time, we performed an anal swab followed by PCR on another subset of mice at 16 weeks postinfection. MmuPV1 was detected in two of eight mice infected and treated with UVB (25%), indicating that a large proportion of FVB/NJ mice cleared the virus by this later time point. Both mice that were found to be positive at 16 weeks postinfection had also been positive at 4 weeks. All infected, non-UVB-treated mice tested again were negative for virus at this later time point (Fig. 4B).

**FIG 5:**
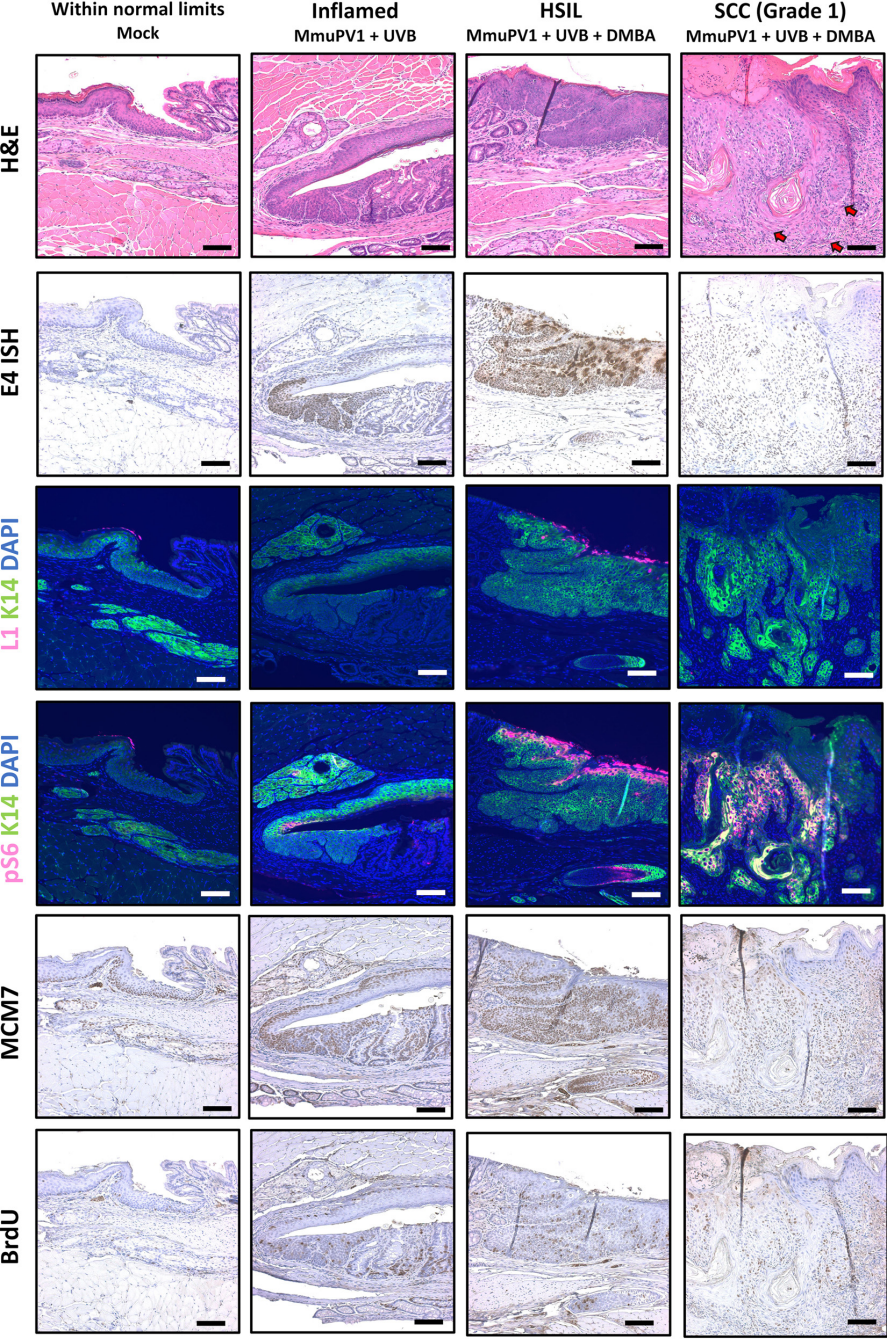
Biomarker analysis of MmuPV1-infected FVB/NJ mice. Representative tissues were stained for markers of viral infection (E4 RNAScope ISH and L1/K14 IF). Based on H&E staining and E4 RNAScope, the representative tissues shown were selected for further analysis using established biomarkers of papillomavirus-mediated disease (pS6/K14 IF, MCM7 IHC) and proliferation (BrdU IHC). All images show tissues stained in the same experiment and were taken with constant exposure for biomarkers of interest. Red arrows within the H&E image mark areas of invasive carcinoma. Figure S6 shows a higher magnification image of the cancer. All scale bars equal 100 μm.

At the 6-month endpoint, anal tissue was harvested and subjected to histopathological grading. In contrast to the high penetrance of high-grade squamous intraepithelial lesions in the MmuPV1-infected NSG mice (Fig. 2C, Table 1), none of the mice infected with MmuPV1 and treated with UVB developed HSIL (Fig. 4C, Table 2). Three of these 13 FVB/NJ mice did develop low-grade squamous intraepithelial lesions, but this was not significantly higher than the state of disease in the mock-infected mice (*P* = 0.11, MmuPV1+UVB versus mock+UVB) (Fig. 4C). Additionally, we were unable to definitively detect virus in these low-grade lesions arising in the infected, UVB-treated FVB/NJ mice via RNAScope ISH (Fig. S2). Of note, at the 6-month postinfection endpoint, MmuPV1 was detected by E4 RNAScope ISH in an area scored as a site of inflammation (Fig. 5), suggesting either that persistence of MmuPV1 in the anal tract of UVB-treated FVB/NJ mice does not always induce dysplasia or that this inflamed region represents a dysplastic lesion actively undergoing regression. Notably, one mouse in our FVB/NJ study as well as one mouse in our NSG carcinogenesis study that were mock infected only progressed to LSIL. This finding is currently unexplained but may be related to inflammation and tissue damage induced during the mock infection (wounding) process.

**TABLE 2:** Disease incidence in FVB/NJ mice, with and without UVB and DMBA treatment

Group	Total no. of mice	Sex^*b*^	Nondysplastic (*n*)	LSIL (*n*)	HSIL (*n*)	SCC grade 1 (*n*)	SCC grade 2 (*n*)
Mock	5	4 M, 1 F	4	1			
Mock + UVB	11	7 M, 4 F	11				
Mock + DMBA	4	0 M, 4 F	2	1	1		
Mock + UVB + DMBA	16	9 M, 7 F	11	3	1	1	
MmuPV1	13	7 M, 6 F	8	4	1		
MmuPV1 + UVB	13	0 M, 13 F	10	3			
MmuPV1 + DMBA	7	5 M, 2 F	4	3			
MmuPV1+ UVB +DMBA^*a*^	15	3 M, 12 F	2	7	2	3	1

a Among MmuPV1+UVB+DMBA mice, the three males were graded as LSIL, HSIL, and SCC grade 2, and there was not a significant difference between males and females in disease severity (*P* = 0.28).

b M, male; F, female.

10.1128/mBio.01611-21.2 FIG S2 Representative lesion showing that viral signal was not definitively detected in LSIL lesions arising in the MmuPV1+UVB group by RNAScope. All scale bars equal 100 μm. Download FIG S2, PDF file, 0.6 MB. Copyright © 2021 Blaine-Sauer et al. 2021 Blaine-Sauer et al. https://creativecommons.org/licenses/by/4.0/ This content is distributed under the terms of the Creative Commons Attribution 4.0 International license.

10.1128/mBio.01611-21.6 FIG S6 Higher magnification image of the cancer shown in Fig. 5, with invasive areas indicated with arrows. Download FIG S6, PDF file, 0.7 MB. Copyright © 2021 Blaine-Sauer et al. 2021 Blaine-Sauer et al. https://creativecommons.org/licenses/by/4.0/ This content is distributed under the terms of the Creative Commons Attribution 4.0 International license.

### MmuPV1 infection, in combination with UVB irradiation and DMBA treatment, contributes to HSIL and cancer in the anal tract of FVB/NJ mice.

Among infected mice irradiated with UVB and topically treated for 20 weeks with DMBA, we observed progression to HSIL and/or the presence of grade 1 or grade 2 invasive squamous cell carcinoma in 40% of the mice (Fig. 4C). The severity of neoplastic disease in the MmuPV1-infected mice was significantly greater than that observed in mock-infected mice that were likewise irradiated with UVB and topically treated for 20 weeks with DMBA (*P* = 0.0036, mmuPV1+DMBA+UVB versus mock+DMBA+UVB) (Fig. 4C), suggesting that MmuPV1 infection cooperates with DMBA to induce high-grade disease and cancer in the anal tract of FVB/NJ mice. To determine if virus was present in the lesions of infected mice at the endpoint, we analyzed tissues for biomarkers of viral infection. Positive staining via RNAScope ISH and immunofluorescence to the L1 capsid protein was observed in an HSIL lesion (Fig. 5), indicating persistent and productive viral infection at the endpoint. However, we were unable to detect the presence of virus within any squamous cell carcinomas via RNAScope or L1 immunofluorescence. To confirm this finding, we isolated DNA from FFPE slide tissues containing the anal squamous cell carcinomas and found that they were negative for MmuPV1 via PCR at the study endpoint (Fig. S3).

10.1128/mBio.01611-21.3 FIG S3 FFPE DNA recovered from tissues harboring invasive squamous cell carcinoma was negative for MmuPV1 by PCR. A mock-infected FVB tissue and HSIL lesion from the MmuPV1+DMBA+UVB group were included as negative and positive controls, respectively. Download FIG S3, PDF file, 0.9 MB. Copyright © 2021 Blaine-Sauer et al. 2021 Blaine-Sauer et al. https://creativecommons.org/licenses/by/4.0/ This content is distributed under the terms of the Creative Commons Attribution 4.0 International license.

MCM7 and pS6 were upregulated within areas of persistent infection with MmuPV1, most notably within the HSIL, with strong viral signal at the endpoint (Fig. 5). We observed a similar staining pattern in the representative HSIL examined in FVB/NJ mice and NSG mice, with strong MCM7 signal detected throughout the full thickness of the lesion and pS6 highly upregulated in the superficial layers of the epithelial lesion. MCM7 was detected within the invasive carcinoma region, although at a lower level than in areas of persistent infection with MmuPV1, whereas pS6 was upregulated in the representative invasive region near the invasive front but not within the upper epithelial layers. BrdU was detected throughout the full thickness in the HSIL, indicating cellular proliferation beyond the basal layer, and more sporadically throughout the invasive carcinoma.

### MmuPV1 persistent infection and disease preferentially localizes to the anal transition zone.

**FIG 6:**
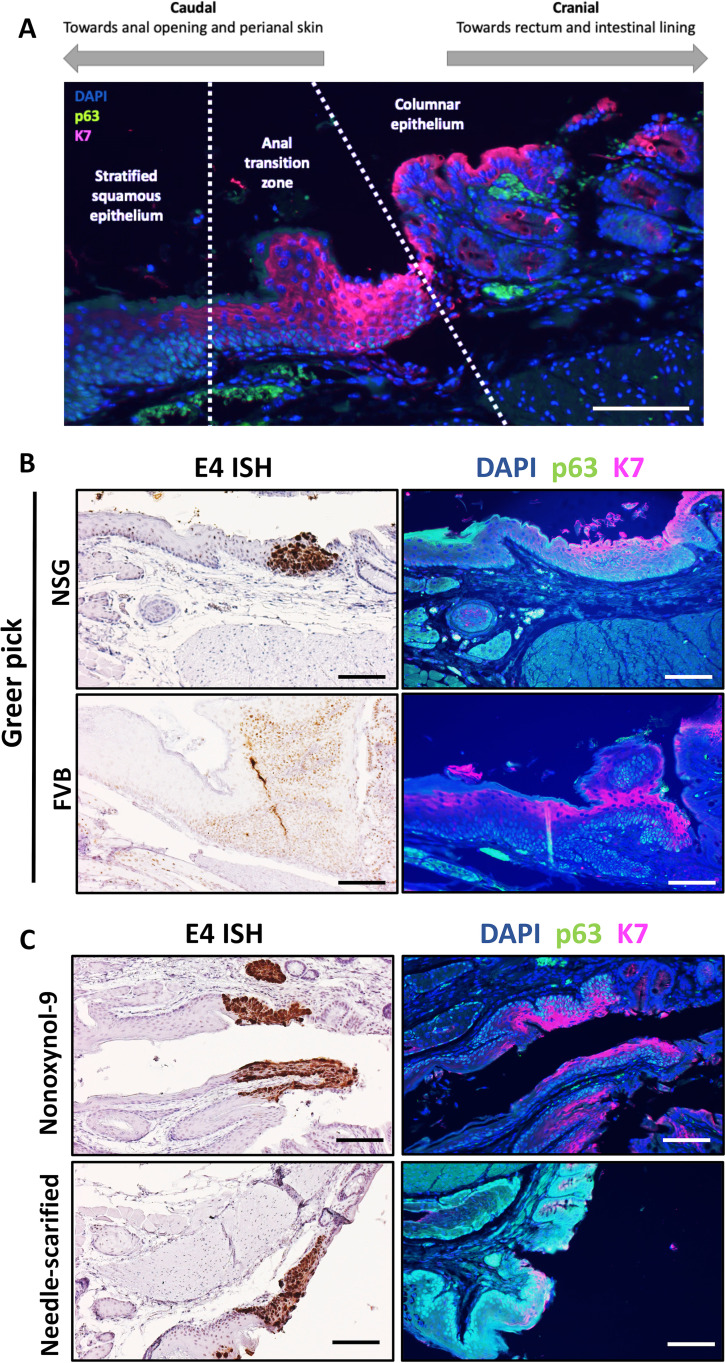
Persistent mouse papillomavirus infection preferentially localizes to the anal transition zone. (A) Microanatomy of the anal transition zone (ATZ). The ATZ is characterized by p63 expression in the nuclei of the basal layer and keratin 7 expression in the suprabasal layers. (B) Areas of persistent infection as shown by RNAScope E4 ISH aligned with the anal transition zone in NSG and FVB/NJ mice infected with the Greer pick. (C) MmuPV1 localized to the anal transition zone in NSG mice infected by two alternative methodologies. All scale bars equal 100 μm.

Based on our initial observation that MmuPV1-mediated disease in NSG mice localized to the anal transition zone, we hypothesized that the transition zone and the stratified squamous epithelium immediately adjacent to it is a preferential site for the establishment of MmuPV1 infection and disease in the anal tract. The anal transition zone is characterized by a distinct pattern of expression of keratin 7 (K7) and p63 (44, 45). Using these biomarkers, the microanatomy of the anal canal can be broken down into three distinct regions: the caudal, stratified squamous epithelium characterized by p63-positive nuclei in the basal layer and K7-negative suprabasal layers, the anal transition zone characterized by K7 expression in the suprabasal layers and p63-positive nuclei in the basal layer, and the columnar epithelium characterized by p63-negative nuclei (Fig. 6A). Using tissues from infected NSG and FVB/NJ mice from our above-described infection studies, we analyzed serial sections of anal tissues positive for MmuPV1 by E4 RNAScope for K7 and p63 expression. We observed that in both NSG and FVB/NJ mice, viral signal was often localized at the anal transition zone, especially areas of high K7 expression (Fig. 6B).

To determine whether the localization of MmuPV1 to the anal transition zone was a biological phenomenon and not an artifact of infection methodology, we experimentally infected NSG mice using two alternative techniques. NSG mice were either chemically wounded using Conceptrol containing 4% nonoxynol-9 or physically wounded by scarifying the anus with a needle. Mice were then infected with virus suspended in 4% carboxyl methylcellulose, which provides viscosity to the solution, aiding in its retention on the surface of the anus. Infection was confirmed via lavage in five of six (83%) chemically wounded mice and seven of seven (100%) needle-scarified mice (Fig. S4). Tissues were collected at 3 months postinfection and analyzed via RNAScope ISH to visualize areas of viral infection as well as p63/K7 dual immunofluorescence to distinguish the anal microanatomical zones. In both the chemically and physically wounded mice, we observed persistent MmuPV1 infections preferentially, but not exclusively, localized at and near the anal transition zone, especially in areas of high K7 expression (Fig. 6C). This result is consistent with the results we obtained with our Greer pick infections and was especially pronounced in the chemically wounded mice. In the needle-scarified tissues, we often observed viral infection within the anal transition zone but also over a wider microanatomical area, including in the more caudal areas toward the perianal skin, possibly due to the severity of the wounding induced throughout the anal epithelium (Fig. S5).

10.1128/mBio.01611-21.4 FIG S4 Lavages of mice chemically wounded with nonyxonol-9 or physically wounded by needle scarification were analyzed for MmuPV1 by PCR, showing that these alternative anal infection methods also efficiently infected NSG mice. Download FIG S4, PDF file, 0.1 MB. Copyright © 2021 Blaine-Sauer et al. 2021 Blaine-Sauer et al. https://creativecommons.org/licenses/by/4.0/ This content is distributed under the terms of the Creative Commons Attribution 4.0 International license.

10.1128/mBio.01611-21.5 FIG S5 Representative tissues showing the full areas of viral infection in alternatively wounded NSG mice at the 3 months study endpoint by E4 RNAScope ISH. All scale bars equal 100 μm. Download FIG S5, PDF file, 0.3 MB. Copyright © 2021 Blaine-Sauer et al. 2021 Blaine-Sauer et al. https://creativecommons.org/licenses/by/4.0/ This content is distributed under the terms of the Creative Commons Attribution 4.0 International license.

## DISCUSSION

In this study, we present a preclinical model for neoplastic anal disease and cancer mediated by a natural infection with MmuPV1. We found that MmuPV1 is able to infect and persist in the anal tract of immunocompromised NSG mice at near 100% efficiency; across all our studies, we failed to detect viral infection of the anus in only one NSG mouse infected with MmuPV1. This mouse was chemically wounded with nonoxynol-9 as part of the alternative infection method (see Fig. S4 in the supplemental material). We hypothesize this was caused by a failure to establish infection due to inefficient wounding; therefore, exposure of the basal epithelium to virus, rather than an infection that regressed. All NSG mice infected using our standard methodology were virus positive at all time points examined. MmuPV1 was originally isolated from cutaneous warts and, thus, was initially hypothesized to be a cutaneous-tropic virus, but our study adds to the growing body of literature showing that the virus is able to infect, persist, and mediate disease in mucosal tissues as well, including the oral cavity and oropharynx, anus, lower female reproductive tract (cervix and vagina), and penis (17, 30, 32, 33, 35–37, 39, 40).

In our studies in immunocompetent mice, we show for the first time that MmuPV1 is able to persist over the course of several months in the anal tract of the FVB/NJ mice when irradiated with UVB. While infections were detected in 71% of these mice at 4 weeks postinfection, subsequent swabbing at 16 weeks and endpoint analysis for viral markers showed that a high percentage of mice went on to clear the MmuPV1 anal infection. In contrast, our group has previously shown that mouse papillomavirus is able to persist over many months in a high percentage of fully immunocompetent FVB/NJ mice without UVB or additional cofactors in the female reproductive tract (33). One possible explanation for the enhanced susceptibility of the cervix to persistent infection compared to the anus is the role of endogenous estrogen. Estrogen has been shown to play a key role in potentiating the development of cervical cancer, and clinical evidence suggests that higher estrogen levels are associated with increased persistence of high-risk HPVs (54, 55). Furthermore, we have shown that MmuPV1 infection persists in 100% of mice treated with exogenous estrogen and that estrogen-treated mice developed more severe disease (33). We conclude that the anal tract is a less permissive site for persistent mouse papillomavirus infection than the female reproductive tract and may be more similar to the oral mucosa in its susceptibility to persistent infection in immunocompetent animals (35), consistent with previous findings tracking MmuPV1 infection in mucosal sites (39). Future studies comprehensively investigating the dynamics and mechanisms of MmuPV1 persistence, immunoevasion, and immunoclearance in the anal tract are warranted.

While MmuPV1 alone induced high-grade squamous intraepithelial lesions in the anal tract of 100% of immunocompromised NSG mice by 6 months postinfection, no mice treated with virus alone progressed to cancer (Fig. 2C, Table 1). By treating with the chemical carcinogen DMBA in addition to MmuPV1 infection in our NSG model, we observed carcinogenic progression, albeit in only one mouse. However, in our NSG study, we treated mice for only 16 weeks with DMBA compared to a 20-week treatment in our FVB/NJ model. Thus, we hypothesize that DMBA treatment over a longer course or at a higher dosage would synergize with MmuPV1 infection to induce a higher frequency of cancers in NSG mice.

In our previously established transgenic model of anal cancer, we observed that the constitutive expression of the HPV oncogenes E6 and E7 from a K14 promoter alone was not sufficient to induce disease or cancer and that treatment with the carcinogen DMBA was necessary (24). Those data, combined with the results of our NSG study, are consistent with the hypothesis that papillomavirus infection alone is insufficient to drive anal carcinogenesis (24) and that it plays a tumor-promoting rather than tumor-initiating role, as we have shown previously for HPV oncogenes (19). Epidemiological studies have shown that despite a high prevalence of anal HPV infections equivalent to or even greater than the infection prevalence in the female reproductive tract in women, the rate of anal carcinogenesis is up to four times lower than that of cervical carcinogenesis (56, 57). The potential cofactors driving the development of anal cancer in combination with persistent HPV infection are not fully understood but may include smoking and systemic inflammation due to Crohn disease, among others (14, 58–62).

In our NSG study, we were unable to definitively detect MmuPV1 signal within the invasive carcinoma of the lone cancer arising in these mice (Fig. 3, Fig. S1). Similarly, in FVB/NJ mice we were unable to detect virus within the invasive carcinomas by either RNAScope or immunofluorescent staining for the L1 capsid protein, and DNA samples recovered from FFPE tissues harboring invasive carcinoma were negative for MmuPV1 by PCR (Fig. 5, Fig. S3). The failure to detect the presence of virus within cancerous lesions at the endpoint of the study raises the possibility that these cancers were DMBA mediated and arose independently of the virus. However, several lines of evidence point to a contributing role for MmuPV1 in carcinogenesis in our studies. First, in our NSG study the difference between MmuPV1+DMBA and mock+DMBA groups was highly significant (*P* = 0.00061), and no NSG mice treated with DMBA alone developed HSIL or cancer by the study endpoint. Second, MmuPV1 nuclear and cytoplasmic signal was detected in areas of the tissue immediately adjacent to the cancer that developed in our NSG mice. This is consistent with previous observations from our group that viral signals are often lower within cancers compared to lower-grade lesions or adjacent noncancerous tissue (33–35). Third, in FVB/NJ mice treated with UVB and DMBA, we detected viral biomarkers at the 6-month endpoint within a high-grade squamous intraepithelial lesion (Fig. 5), suggesting MmuPV1 plays a contributing role in progression to high-grade precancerous disease even in immunocompetent mice. Finally, we observed a highly significant difference in disease severity in FVB/NJ mice between MmuPV1-infected mice treated with UVB and DMBA and their mock-infected counterparts (*P* = 0.0036), with only 1 of 16 (6%) mock+UVB+DMBA mice developing squamous cell carcinoma compared to 4 of 15 (27%) MmuPV1+UVB+DMBA mice. Taken together, our data suggest that MmuPV1 contributes to carcinogenesis even when undetected in cancerous lesions at the endpoint. A “hit-and-run” role of papillomaviruses in carcinogenesis is supported by several previous studies but remains controversial (63–65). A related hypothesis is that MmuPV1 infection creates a microenvironment with an expanded pool of epithelial cells susceptible to chemically induced carcinogenesis by DMBA, as suggested by N. Christensen during review of the manuscript. Further studies distinguishing the roles of MmuPV1 and DMBA at different stages of anal disease and carcinogenesis are warranted.

A key contribution of our study is the finding that MmuPV1 infection preferentially localizes to the area at and around the anal transition zone (Fig. 6), which has previously been shown to be susceptible to MmuPV1 infection (28, 40). We robustly demonstrate this localization effect by comparing viral infection biomarkers to p63 and K7 biomarkers characterizing the anal microanatomical zones (44, 45). Notably, we find that areas of persistent MmuPV1 infection often correspond to areas with high levels of K7 expression in the suprabasal layers marking the transition zone, and that this effect is independent of infection methodology. Interestingly, while in humans a K7-positive squamocolumnar transitional region is associated with increased susceptibility to HPV infection in the cervix (66), the K7-positive anal transition zone appears comparatively less susceptible to papillomavirus infection, with only 27% of examined anal lesions in one study originating at the anal transition zone (44). Similarly, the significance of the K7-positive transitional epithelium in human head and neck cancers and its susceptibility to HPV infection is less clearly defined than in the cervix, although K7 has been shown to be associated with HPV-positive oropharyngeal cancers (67, 68). Keratin 7 has also been shown to stabilize HPV16 E7 transcripts via the SEQIKA peptide region, although no similar analysis has been performed for MmuPV1 (69). Further study is warranted into the role that the transitional microanatomy and K7 specifically may play in papillomavirus infections at different sites in both mice and humans. It will also be of interest to study perianal disease using MmuPV1.

Recently, J. M. Palefsky and colleagues successfully generated an HPV16-transformed human anal epithelial cell line, a first in the field, providing an *in vitro* preclinical platform for studies on anal carcinogenesis. This cell line displays poor terminal differentiation properties and an ability to invade, and it forms colonies when suspended in soft agar (70). They discovered that the HPV16 E5 gene, which is found to be expressed in human anal cancers, contributed to the transformed phenotype of this HPV16-positive anal epithelial cell line. MmuPV1 does not encode an E5 gene. We discovered that the HPV16 E5 gene, as expressed in K14E5 transgenic FVB/NJ mice, enhanced the ability of MmuPV1 to cause pathogenesis of the mouse skin, leading to larger lesions that were less likely to regress and were more likely to progress to SCC than in nontransgenic FVB/NJ mice (41). E5 also enhanced the ability of MmuPV1 to cause cancers of the female reproductive tract in this study. Thus, it may be of interest to do parallel studies with K14E5 transgenic mice in the context of our model of MmuPV1-mediated anal disease to determine if E5 contributes to increased persistence of MmuPV1 and/or increased anal carcinogenesis and whether cancers arising in the K14E5 mice retain MmuPV1.

In summary, we show for the first time that MmuPV1 infection alone efficiently mediates high-grade squamous intraepithelial lesions in the anal tract of NSG mice and that MmuPV1 has carcinogenic potential in combination with DMBA. Furthermore, we demonstrate that MmuPV1 is able to infect and persist for up to 6 months in the anal tract of fully immunocompetent FVB/NJ mice when irradiated with UVB and contributes to HSIL and invasive carcinoma development with the addition of DMBA. We also show that persistent MmuPV1 infection preferentially localizes at and around the anal transition zone, as defined by biomarker analysis, and that this effect is not an artifact of infection methodology. This novel model of anal disease and cancer driven by a natural infection provides a platform for future studies investigating the mechanisms of papillomavirus-mediated disease and a valuable preclinical model for evaluating antiviral and anticancer therapies.

## MATERIALS AND METHODS

### Mice.

NOD scid gamma (NSG) mice were purchased from Jackson Laboratory (stock number 005557) and bred by the UW-Madison Biomedical Research Models Services Laboratory. All NSG mice were infected at 8 to 10 weeks of age and were maintained under aseptic conditions. FVB/NJ mice were purchased from Taconic, bred for our studies, and infected at 4 to 6 weeks of age. All mice were housed in the University of Wisconsin School of Medicine and Public Health, Association for Assessment of Laboratory Animal Care-approved, Animal Care Unit. All procedures were carried out in accordance with an animal protocol M005871 approved by the University of Wisconsin School of Medicine and Public Health Institutional Animal Care and Use Committee.

### Infection of the anal tract with MmuPV1.

Infections of MmuPV1 in the initial NSG infection study, the NSG carcinogenesis study, and the FVB/NJ study were carried out using a Greer pick (Greer Laboratories, Inc., Lenoir, NC) allergen skin-testing device. Mice were anesthetized with isoflurane, and the anus was dilated manually with forceps to allow access for the pick to the target tissue. MmuPV1 virus stock was generated from the warts of *FoxN1^nu/nu^* mice as previously described (34). The Greer pick was dipped in 3 × 10^8^ viral genome equivalents (VGE) per μl stock, suspending ∼1.1 μl within the pick via capillary action. The pick was then inserted into the anal tract of the mice, targeting ∼2 to 5 mm into the anal mucosa, wounding with the teeth of the pick by pressing firmly into the mucosa and discharging the virus onto the exposed area. This was performed once each on both the dorsal and ventral sides of the anal canal for all studies using NSG mice and on the dorsal side only for studies in FVB/NJ mice. Mock infections were performed using the same technique with PBS only.

NSG mice were also infected using two alternative infection methods. In the first alternative method, mice were chemically wounded using a method adopted from our group’s cervicovaginal infection protocol using MmuPV1 (33). Briefly, 50 μl Conceptrol (247149; Options), a spermicidal gel containing 4% nonoxynol-9, was injected into the anal canal. Four hours later, mice were infected with 10^8^ VGE of MmuPV1 suspended in 25 μl 4% carboxyl methylcellulose (CMC) (C4888; Sigma). In the second alternative method, mice were wounded by inserting an 18-gauge needle ∼1 cm into the anus and abrading the anal mucosa by applying light pressure as the needle was pulled out slowly, with the bevel facing away from the tissue. This was performed five times each for both the ventral and dorsal sides of the anal canal for each mouse. Mice were then immediately infected with 10^8^ VGE of MmuPV1 suspended in 25 μl 4% CMC or 2 × 10^7^ VGE of MmuPV1 suspended in 5 μl 4% CMC. For both alternative infection methods, mock infections were performed using the same techniques but treating with 4% CMC only.

### UVB irradiation.

FVB/NJ mice were exposed to a single dose of 1,000 mJ/cm^2^ UVB spectral irradiation at 24 h postinfection as previously described using a custom-built unit (Daavlin, Bryan, OH) (34, 71).

### DMBA treatment.

Dimethylbenz(a)anthracene (DMBA) was dissolved in 60% acetone–40% dimethyl sulfoxide (DMSO) and was administered topically one time per week at a dose of 0.12 μmol as previously described (22). The acetone-DMSO vehicle was administered similarly as a control. DMBA or vehicle was administered for 20 weeks beginning 1 week postinfection in the FVB/NJ study and for 16 weeks beginning at week 10 postinfection in the NSG carcinogenesis study.

### Tissue collection and histological grading.

Anal tracts were harvested, fixed in 4% paraformaldehyde for 24 h, processed, bisected, and embedded cut-side down in paraffin. Five-micrometer serial sections were cut, and every 20th section was stained by H&E. Blinded histopathological scoring was performed by Kristina Matkowskyj, a trained gastrointestinal pathologist in the UW SMPH Department of Pathology and Laboratory Medicine. Intraepithelial lesions were graded consistent with the current LAST criteria (72), and cancers were graded using a 3-tier system based on the degree to which the invasive component resembled its site of origin (well, moderately, or poorly differentiated). The highest grade of disease severity observed within each tissue is reported.

### Detection of MmuPV1 by anal lavage or swab and PCR.

Anal swabs were performed by prewetting a cotton swab (Fenshine; Microblading Cotton Swab) in PBS, swabbing the interior of the anal canal, and then soaking the pick in 200 μl PBS. Lavages were performed using a method adopted from Hu et al. (39) by pipetting 25 μl PBS into the anal tract, triturating several times and collecting, and then bringing the total sample volume to 200 μl with PBS. Samples were frozen at −20°C, and DNA extraction was performed using the DNeasy blood and tissue kit (69506; Qiagen) by following the manufacturer’s protocol. DNA was subsequently amplified via PCR with primers specific to the MmuPV1 genome in either the E2 region (F, 5′-GCCCGAAGACAACACCGCCACG-3′; R, 5′-CCTCCGCCTCGTCCCCAAATGG-3′) or the L1 region (F, 5′-GGAAGGAGAGAGCAAGTGTATG-3′; R, 5′-GGGTTTGGTGTGTTGGTTTG-3′) and analyzed via agarose gel.

### DNA recovery from FFPE tissues.

Tissues from two slides per sample (4 total tissue sections) were scraped into Eppendorf tubes and incubated in xylene at room temperature for 5 min. Samples were centrifuged, supernatant was removed, and the xylene incubation was repeated. Samples were then washed twice with 100% ethanol and air dried; 200 μl 0.5% Tween 20 in Tris-EDTA, pH 8.0, and 200 μg proteinase K were added, and samples were incubated at 55°C for 3 h. Samples were boiled for 5 min and then ethanol precipitated with 0.1 volume 3 M NaOAc and 2.5 volumes 100% ethanol for 30 min at −20°C. Samples were centrifuged at maximum for 15 min at 4°C, and pellets were washed with 500 μl 70% ethanol, air dried, and resuspended in 30 μl double-distilled water. PCR was performed for MmuPV1 as described above.

### BrdU incorporation.

One hour prior to sacrifice, mice were injected intraperitoneally with 300 μl of 12.5 mg/ml BrdU (203806; Calbiochem) dissolved in PBS. Tissues were processed, and BrdU was detected by immunohistochemistry (IHC).

### Immunohistochemistry.

BrdU and MCM7 were detected using the M.O.M. ImmPRESS kit (P-2400; Vector) according to the manufacturer’s instructions. BrdU mouse monoclonal antibody (MAb) (1:50) (NA61; Calbiochem) and MCM7 mouse MAb (1:200) (Neomarkers [ms-862-p] or Invitrogen [MA5-14291]) were incubated overnight at 4°C. Dual p63/K7 staining was performed with antigen retrieval in 10 mM citrate buffer. p63 mouse MAb (MAB4135; Millipore) and K7 rabbit polyclonal antibody (pAb; 15539-1-AP; Proteintech) were incubated at 4°C overnight at 1:100 and detected with fluorescent Alexa Flour secondary antibodies. Dual L1/K14 or pS6/K14 tyramide signal amplification immunofluorescence was performed as previously described (41) using L1 rabbit pAb (1:5,000) provided by Chris Buck (National Institutes of Health), pS6 (Ser235/236) rabbit MAb (1:4,000) (4858; Cell Signaling), and K14 rabbit pAb (1:1,000) (905301; BioLegend).

### RNAScope *in situ* hybridization.

Detection of MmuPV1 viral signal was performed using the RNAScope 2.5 HD assay-brown kit (322300; Advanced Cell Diagnostics, Newark, CA) according to the manufacturer's protocol using a probe to MmuPV1 E4 (473281) or MmuPV1 E6/E7 (409771). As controls, select tissues were treated with 20 U of DNase I (EN0521; Thermo Fisher), 500 μg RNase A (1006657; Qiagen), and 2,000 U RNase T1 (EN0542; Thermo Fisher) for 30 min at 40°C prior to probing.

### MmuPV1 FISH.

FISH for MmuPV1 DNA was performed on processed tissues as previously described using a nick-translated digoxigenin-labeled probe (73).

### Image acquisition.

All images were acquired using a Zeiss AxioImager M2 microscope with AxioVision software.

### Statistical analysis.

Comparisons of disease severity between groups were analyzed by assigning each histopathological grade a rank (1, nondysplastic; 2, LSIL; 3, HSIL; 4, SCC grade 1; 5, SCC grade 2). Ranks were then analyzed by two-sided Wilcoxon rank sum test using the calculator at https://astatsa.com/WilcoxonTest/. A *P* value of less than 0.05 was considered significant. A *P* value of less than 0.01 was considered highly significant.

## References

[1] Siegel RL, Miller KD, Fuchs HE, Jemal A. 2021. Cancer statistics, 2021. Cancer J Clin 71:7–33. doi:10.3322/caac.21654. 33433946

[2] Nelson RA, Levine AM, Bernstein L, Smith DD, Lai LL. 2013. Changing patterns of anal canal carcinoma in the United States. J Clin Oncol 31:1569–1575. doi:10.1200/JCO.2012.45.2524. 23509304 PMC3753461

[3] Deshmukh AA, Suk R, Shiels MS, Sonawane K, Nyitray AG, Liu Y, Gaisa MM, Palefsky JM, Sigel K. 2020. Recent Trends in Squamous Cell Carcinoma of the Anus Incidence and Mortality in the United States, 2001–2015. J Natl Cancer Inst 112:829–838. doi:10.1093/jnci/djz219. 31742639 PMC7825484

[4] Shridhar R, Shibata D, Chan E, Thomas CR. 2015. Anal cancer: current standards in care and recent changes in practice. Cancer J Clin 65:139–162. doi:10.3322/caac.21259. 25582527

[5] Bentzen AG, Balteskard L, Wanderås EH, Frykholm G, Wilsgaard T, Dahl O, Guren MG. 2013. Impaired health-related quality of life after chemoradiotherapy for anal cancer: late effects in a national cohort of 128 survivors. Acta Oncol 52:736–744. doi:10.3109/0284186X.2013.770599. 23438358

[6] Allal AS, Sprangers MA, Laurencet F, Reymond MA, Kurtz JM. 1999. Assessment of long-term quality of life in patients with anal carcinomas treated by radiotherapy with or without chemotherapy. Br J Cancer 80:1588–1594. doi:10.1038/sj.bjc.6690567. 10408404 PMC2363100

[7] Daling JR, Madeleine MM, Johnson LG, Schwartz SM, Shera KA, Wurscher MA, Carter JJ, Porter PL, Galloway DA, McDougall JK. 2004. Human papillomavirus, smoking, and sexual practices in the etiology of anal cancer. Cancer 101:270–280. doi:10.1002/cncr.20365. 15241823

[8] Baricevic I, He X, Chakrabarty B, Oliver AW, Bailey C, Summers J, Hampson L, Hampson I, Gilbert DC, Renehan AG. 2015. High-sensitivity human papilloma virus genotyping reveals near universal positivity in anal squamous cell carcinoma: different implications for vaccine prevention and prognosis. Eur J Cancer 51:776–785. doi:10.1016/j.ejca.2015.01.058. 25702585

[9] Saraiya M, Unger ER, Thompson TD, Lynch CF, Hernandez BY, Lyu CW, Steinau M, Watson M, Wilkinson EJ, Hopenhayn C, Copeland G, Cozen W, Peters ES, Huang Y, Saber MS, Altekruse S, Goodman MT, HPV Typing of Cancers Workgroup. 2015. US assessment of HPV types in cancers: implications for current and 9-valent HPV vaccines. J Natl Cancer Inst 107:djv086. doi:10.1093/jnci/djv086. 25925419 PMC4838063

[10] De Vuyst H, Clifford GM, Nascimento MC, Madeleine MM, Franceschi S. 2009. Prevalence and type distribution of human papillomavirus in carcinoma and intraepithelial neoplasia of the vulva, vagina and anus: a meta-analysis. Int J Cancer 124:1626–1636. doi:10.1002/ijc.24116. 19115209

[11] Colón-López V, Shiels MS, Machin M, Ortiz AP, Strickler H, Castle PE, Pfeiffer RM, Engels EA. 2018. Anal cancer risk among people with HIV infection in the United States. J Clin Oncol 36:68–75. doi:10.1200/JCO.2017.74.9291. 29140774 PMC5791846

[12] Albuquerque A, Stirrup O, Nathan M, Clifford GM. 2020. Burden of anal squamous cell carcinoma, squamous intraepithelial lesions and HPV16 infection in solid organ transplant recipients: a systematic review and meta-analysis. Am J Transplant 20:3520–3528. doi:10.1111/ajt.15942. 32343489

[13] Tseng H-F, Morgenstern H, Mack TM, Peters RK. 2003. Risk factors for anal cancer: results of a population-based case–control study. Cancer Causes Control 14:837–846. doi:10.1023/b:caco.0000003837.10664.7f. 14682441

[14] Grulich AE, Poynten IM, Machalek DA, Jin F, Templeton DJ, Hillman RJ. 2012. The epidemiology of anal cancer. Sex Health 9:504–508. doi:10.1071/SH12070. 22958581

[15] Tilston P. 1997. Anal human papillomavirus and anal cancer. J Clin Pathol 50:625–634. doi:10.1136/jcp.50.8.625. 9301544 PMC500098

[16] Joseph DA, Miller JW, Wu X, Chen VW, Morris CR, Goodman MT, Villalon-Gomez JM, Williams MA, Cress RD. 2008. Understanding the burden of human papillomavirus-associated anal cancers in the US. Cancer 113:2892–2900. doi:10.1002/cncr.23744. 18980293 PMC2729501

[17] Ingle A, Ghim S, Joh J, Chepkoech I, Bennett Jenson A, Sundberg JP. 2011. Novel laboratory mouse papillomavirus (MusPV) infection. Vet Pathol 48:500–505. doi:10.1177/0300985810377186. 20685915

[18] Eckert RL, Crish JF, Balasubramanian S, Rorke EA. 2000. Transgenic animal models of human papillomavirus-dependent disease (review). Int J Oncol 16:853–870. 10762621

[19] Song S, Liem A, Miller JA, Lambert PF. 2000. Human papillomavirus types 16 E6 and E7 contribute differently to carcinogenesis. Virology 267:141–150. doi:10.1006/viro.1999.0106. 10662610

[20] Thomas MK, Pitot HC, Liem A, Lambert PF. 2011. Dominant role of HPV16 E7 in anal carcinogenesis. Virology 421:114–118. doi:10.1016/j.virol.2011.09.018. 21999991 PMC3258583

[21] Carchman EH, Matkowskyj KA, Meske L, Lambert PF. 2016. Dysregulation of autophagy contributes to anal carcinogenesis. PLoS One 11:e0164273. doi:10.1371/journal.pone.0164273. 27706233 PMC5051741

[22] Stelzer MK, Pitot HC, Liem A, Lee D, Kennedy GD, Lambert PF. 2010. Rapamycin inhibits anal carcinogenesis in two preclinical animal models. Cancer Prev Res 3:1542–1551. doi:10.1158/1940-6207.CAPR-10-0228. PMC3058330 21149330

[23] Shin M-K, Payne S, Bilger A, Matkowskyj KA, Carchman E, Meyer DS, Bentires-Alj M, Deming DA, Lambert PF. 2019. Activating mutations in Pik3ca contribute to anal carcinogenesis in the presence or absence of HPV-16 oncogenes. Clin Cancer Res 25:1889–1900. doi:10.1158/1078-0432.CCR-18-2843. 30530704 PMC6423984

[24] Stelzer MK, Pitot HC, Liem A, Schweizer J, Mahoney C, Lambert PF. 2010. A mouse model for human anal cancer. Cancer Prev Res 3:1534–1541. doi:10.1158/1940-6207.CAPR-10-0086. PMC3006089 20947489

[25] Rademacher BL, Matkowskyj KA, Meske LM, Romero A, Sleiman H, Carchman EH. 2017. The role of pharmacologic modulation of autophagy on anal cancer development in an HPV mouse model of carcinogenesis. Virology 507:82–88. doi:10.1016/j.virol.2017.04.007. 28431282 PMC5584602

[26] Rademacher BL, Matkowskyj KA, LaCount ED, Carchman EH. 2019. Topical application of a dual PI3K/mTOR inhibitor prevents anal carcinogenesis in a human papillomavirus mouse model of anal cancer. Eur J Cancer Prev 28:483–491. doi:10.1097/CEJ.0000000000000505. 30888976 PMC6751036

[27] Rademacher BL, Meske LM, Matkowskyj KA, Hanlon BM, Carchman EH. 2018. Genetic inhibition of autophagy in a transgenic mouse model of anal cancer. J Carcinog 17:3. doi:10.4103/jcar.JCar_4_18. 30123096 PMC6071480

[28] Hu J, Cladel NM, Budgeon LR, Balogh KK, Christensen ND. 2017. The mouse papillomavirus infection model. Viruses 9:246. doi:10.3390/v9090246. PMC5618012 28867783

[29] Uberoi A, Lambert PF. 2017. Rodent papillomaviruses. Viruses 9:362. doi:10.3390/v9120362. PMC5744137 29186900

[30] Spurgeon ME, Lambert PF. 2019. Sexual transmission of murine papillomavirus (MmuPV1) in Mus musculus. Elife 8:e50056. doi:10.7554/eLife.50056. 31621578 PMC6797482

[31] Cladel NM, Budgeon LR, Cooper TK, Balogh KK, Christensen ND, Myers R, Majerciak V, Gotte D, Zheng Z-M, Hu J. 2017. Mouse papillomavirus infections spread to cutaneous sites with progression to malignancy. J Gen Virol 98:2520–2529. doi:10.1099/jgv.0.000926. 28942760 PMC5845569

[32] Cladel NM, Budgeon LR, Balogh KK, Cooper TK, Brendle SA, Christensen ND, Schell TD, Hu J. 2017. Mouse papillomavirus infection persists in mucosal tissues of an immunocompetent mouse strain and progresses to cancer. Sci Rep 7:16932. doi:10.1038/s41598-017-17089-4. 29208932 PMC5717108

[33] Spurgeon ME, Uberoi A, McGregor SM, Wei T, Ward-Shaw E, Lambert PF. 2019. A novel in vivo infection model to study papillomavirus-mediated disease of the female reproductive tract. mBio 10:e00180-19. doi:10.1128/mBio.00180-19. 30837335 PMC6401479

[34] Uberoi A, Yoshida S, Frazer IH, Pitot HC, Lambert PF. 2016. Role of ultraviolet radiation in papillomavirus-induced disease. PLoS Pathog 12:e1005664. doi:10.1371/journal.ppat.1005664. 27244228 PMC4887022

[35] Wei T, Buehler D, Ward-Shaw E, Lambert PF. 2020. An infection-based murine model for papillomavirus-associated head and neck cancer. mBio 11:e00908-20. doi:10.1128/mBio.00908-20. 32398315 PMC7218285

[36] Spurgeon ME, Lambert PF. 2020. Mus musculus papillomavirus 1: a new frontier in animal models of papillomavirus pathogenesis. J Virol 94:e00002-20. doi:10.1128/JVI.00002-20. 32051276 PMC7163119

[37] Bilger A, King RE, Schroeder JP, Piette JT, Hinshaw LA, Kurth AD, AlRamahi RW, Barthel MV, Ward-Shaw ET, Buehler D, Masters KS, Thibeault SL, Lambert PF. 2020. A mouse model of oropharyngeal papillomavirus-induced neoplasia using novel tools for infection and nasal anesthesia. Viruses 12:v12040450. doi:10.3390/v12040450. PMC7232375 32316091

[38] Cladel NM, Budgeon LR, Cooper TK, Balogh KK, Hu J, Christensen ND. 2013. Secondary infections, expanded tissue tropism, and evidence for malignant potential in immunocompromised mice infected with Mus musculus papillomavirus 1 DNA and virus. J Virol 87:9391–9395. doi:10.1128/JVI.00777-13. 23785210 PMC3754027

[39] Hu J, Budgeon LR, Cladel NM, Balogh K, Myers R, Cooper TK, Christensen ND. 2015. Tracking vaginal, anal and oral infection in a mouse papillomavirus infection model. J Gen Virol 96:3554–3565. doi:10.1099/jgv.0.000295. 26399579 PMC4804763

[40] Cladel NM, Budgeon LR, Balogh KK, Cooper TK, Hu J, Christensen ND. 2015. A novel pre-clinical murine model to study the life cycle and progression of cervical and anal papillomavirus infections. PLoS One 10:e0120128. doi:10.1371/journal.pone.0120128. 25803616 PMC4372414

[41] Torres AD, Spurgeon ME, Bilger A, Blaine-Sauer S, Uberoi A, Buehler D, McGregor SM, Ward-Shaw E, Lambert PF. 2020. The human papillomavirus 16 E5 gene potentiates MmuPV1-dependent pathogenesis. Virology 541:1–12. doi:10.1016/j.virol.2019.12.002. 31826841 PMC7024000

[42] Ito M, Hiramatsu H, Kobayashi K, Suzue K, Kawahata M, Hioki K, Ueyama Y, Koyanagi Y, Sugamura K, Tsuji K, Heike T, Nakahata T. 2002. NOD/SCID/gamma(c)(null) mouse: an excellent recipient mouse model for engraftment of human cells. Blood 100:3175–3182. doi:10.1182/blood-2001-12-0207. 12384415

[43] Pyeon D, Pearce SM, Lank SM, Ahlquist P, Lambert PF. 2009. Establishment of human papillomavirus infection requires cell cycle progression. PLoS Pathog 5:e1000318. doi:10.1371/journal.ppat.1000318. 19247434 PMC2642596

[44] Yang EJ, Quick MC, Hanamornroongruang S, Lai K, Doyle LA, McKeon FD, Xian W, Crum CP, Herfs M. 2015. Microanatomy of the cervical and anorectal squamocolumnar junctions: a proposed model for anatomical differences in HPV-related cancer risk. Mod Pathol 28:994–1000. doi:10.1038/modpathol.2015.54. 25975286 PMC4490106

[45] Clavero O, McCloskey J, Molina VM, Quirós B, Bravo IG, de Sanjosé S, Bosch FX, Pimenoff VN. 2017. Squamous intraepithelial lesions of the anal squamocolumnar junction: histopathological classification and HPV genotyping. Papillomavirus Res 3:11–17. doi:10.1016/j.pvr.2016.12.001. 28720443 PMC5883205

[46] Zhou Y, Pan Y, Zhang S, Shi X, Ning T, Ke Y. 2007. Increased phosphorylation of p70 S6 kinase is associated with HPV16 infection in cervical cancer and esophageal cancer. Br J Cancer 97:218–222. doi:10.1038/sj.bjc.6603838. 17622239 PMC2360306

[47] Hernandez J, Elahi A, Siegel E, Coppola D, Riggs B, Shibata D. 2011. HPV L1 capsid protein detection and progression of anal squamous neoplasia. Am J Clin Pathol 135:436–441. doi:10.1309/AJCPR5VD6NSQRWBN. 21350099 PMC4511275

[48] Melsheimer P, Kaul S, Dobeck S, Bastert G. 2003. Immunocytochemical detection of HPV high-risk type L1 capsid proteins in LSIL and HSIL as compared with detection of HPV L1 DNA. Acta Cytol 47:124–128. doi:10.1159/000326491. 12685176

[49] Yoshida T, Sano T, Kanuma T, Owada N, Sakurai S, Fukuda T, Nakajima T. 2008. Immunochemical analysis of HPV L1 capsid protein and p16 protein in liquid-based cytology samples from uterine cervical lesions. Cancer 114:83–88. doi:10.1002/cncr.23366. 18300235

[50] Lee H, Lee K-J, Jung C-K, Hong J-H, Lee Y-S, Choi Y-J, Lee K-Y, Park G. 2008. Expression of HPV L1 capsid protein in cervical specimens with HPV infection. Diagn Cytopathol 36:864–867. doi:10.1002/dc.20922. 18855883

[51] Middleton K, Peh W, Southern S, Griffin H, Sotlar K, Nakahara T, El-Sherif A, Morris L, Seth R, Hibma M, Jenkins D, Lambert P, Coleman N, Doorbar J. 2003. Organization of human papillomavirus productive cycle during neoplastic progression provides a basis for selection of diagnostic markers. J Virol 77:10186–10201. doi:10.1128/jvi.77.19.10186-10201.2003. 12970404 PMC228472

[52] Xue X-Y, Majerciak V, Uberoi A, Kim B-H, Gotte D, Chen X, Cam M, Lambert PF, Zheng Z-M. 2017. The full transcription map of mouse papillomavirus type 1 (MmuPV1) in mouse wart tissues. PLoS Pathog 13:e1006715. doi:10.1371/journal.ppat.1006715. 29176795 PMC5720830

[53] Strati K, Pitot HC, Lambert PF. 2006. Identification of biomarkers that distinguish human papillomavirus (HPV)-positive versus HPV-negative head and neck cancers in a mouse model. Proc Natl Acad Sci U S A 103:14152–14157. doi:10.1073/pnas.0606698103. 16959885 PMC1599927

[54] Ding L, Liu C, Zhou Q, Feng M, Wang J. 2019. Association of estradiol and HPV/HPV16 infection with the occurrence of cervical squamous cell carcinoma. Oncol Lett 17:3548–3554. doi:10.3892/ol.2019.10005. 30867796 PMC6396129

[55] Chung S-H, Franceschi S, Lambert PF. 2010. Estrogen and ERalpha: culprits in cervical cancer? Trends Endocrinol Metab 21:504–511. doi:10.1016/j.tem.2010.03.005. 20456973 PMC2914219

[56] Stier EA, Sebring MC, Mendez AE, Ba FS, Trimble DD, Chiao EY. 2015. Prevalence of anal human papillomavirus infection and anal HPV-related disorders in women: a systematic review. Am J Obstet Gynecol 213:278–309. doi:10.1016/j.ajog.2015.03.034. 25797230 PMC4556545

[57] Moscicki A-B, Darragh TM, Berry-Lawhorn JM, Roberts JM, Khan MJ, Boardman LA, Chiao E, Einstein MH, Goldstone SE, Jay N, Likes WM, Stier EA, Welton ML, Wiley DJ, Palefsky JM. 2015. Screening for anal cancer in women. J Low Genit Tract Dis 19:S27–42. doi:10.1097/LGT.0000000000000117. 26103446 PMC4479419

[58] Slater G, Greenstein A, Aufses AH. 1984. Anal carcinoma in patients with Crohn’s disease. Ann Surg 199:348–350. doi:10.1097/00000658-198403000-00016. 6703795 PMC1353403

[59] Coffey K, Beral V, Green J, Reeves G, Barnes I, Million Women Study Collaborators. 2015. Lifestyle and reproductive risk factors associated with anal cancer in women aged over 50 years. Br J Cancer 112:1568–1574. doi:10.1038/bjc.2015.89. 25867258 PMC4453684

[60] Vuitton L, Jacquin E, Parmentier A-L, Crochet E, Fein F, Dupont-Gossart A-C, Plastaras L, Bretagne C-H, Mauny F, Koch S, Prétet J-L, Mougin C, Valmary-Degano S. 2018. High prevalence of anal canal high-risk human papillomavirus infection in patients with Crohn’s disease. Clin Gastroenterol Hepatol 16:1768–1776. doi:10.1016/j.cgh.2018.03.008. 29551740

[61] Beaugerie L, Carrat F, Nahon S, Zeitoun J-D, Sabaté J-M, Peyrin-Biroulet L, Colombel J-F, Allez M, Fléjou J-F, Kirchgesner J, Svrcek M, Cancers et Surrisque Associé aux Maladies Inflammatoires Intestinales En France Study Group. 2018. High risk of anal and rectal cancer in patients with anal and/or perianal Crohn’s disease. Clin Gastroenterol Hepatol 16:892–899. doi:10.1016/j.cgh.2017.11.041. 29199142

[62] Lerman J, Hennequin C, Etienney I, Abramowitz L, Goujon G, Gornet J-M, Guillerm S, Aparicio T, Valverde A, Cattan P, Quéro L. 2020. Impact of tobacco smoking on the patient’s outcome after (chemo)radiotherapy for anal cancer. Eur J Cancer 141:143–151. doi:10.1016/j.ejca.2020.09.039. 33137590

[63] Iwasaka T, Hayashi Y, Yokoyama M, Hara K, Matsuo N, Sugimori H. 1992. Hit and run oncogenesis by human papillomavirus type 18 DNA. Acta Obstet Gynecol Scand 71:219–223. doi:10.3109/00016349209009922. 1317646

[64] Niller HH, Wolf H, Minarovits J. 2011. Viral hit and run-oncogenesis: genetic and epigenetic scenarios. Cancer Lett 305:200–217. doi:10.1016/j.canlet.2010.08.007. 20813452

[65] Viarisio D, Müller-Decker K, Accardi R, Robitaille A, Dürst M, Beer K, Jansen L, Flechtenmacher C, Bozza M, Harbottle R, Voegele C, Ardin M, Zavadil J, Caldeira S, Gissmann L, Tommasino M. 2018. Beta HPV38 oncoproteins act with a hit-and-run mechanism in ultraviolet radiation-induced skin carcinogenesis in mice. PLoS Pathog 14:e1006783. doi:10.1371/journal.ppat.1006783. 29324843 PMC5764406

[66] Herfs M, Yamamoto Y, Laury A, Wang X, Nucci MR, McLaughlin-Drubin ME, Münger K, Feldman S, McKeon FD, Xian W, Crum CP. 2012. A discrete population of squamocolumnar junction cells implicated in the pathogenesis of cervical cancer. Proc Natl Acad Sci U S A 109:10516–10521. doi:10.1073/pnas.1202684109. 22689991 PMC3387104

[67] Mehrad M, Dupont WD, Plummer WD, Lewis JS. 2018. Expression and significance of cytokeratin 7, a squamocolumnar junction marker, in head and neck squamous cell carcinoma. Head Neck Pathol 12:448–454. doi:10.1007/s12105-017-0874-2. 29235037 PMC6232215

[68] Woods RSR, Keegan H, White C, Tewari P, Toner M, Kennedy S, O'Regan EM, Martin CM, Timon CVI, O'Leary JJ. 2017. Cytokeratin 7 in oropharyngeal squamous cell carcinoma: a junctional biomarker for human papillomavirus-related tumors. Cancer Epidemiol Biomarkers Prev 26:702–710. doi:10.1158/1055-9965.EPI-16-0619. 28082347

[69] Kanduc D. 2002. Translational regulation of human papillomavirus type 16 E7 mRNA by the peptide SEQIKA, shared by rabbit alpha(1)-globin and human cytokeratin 7. J Virol 76:7040–7048. doi:10.1128/jvi.76.14.7040-7048.2002. 12072504 PMC136328

[70] Wechsler EI, Tugizov S, Herrera R, Da Costa M, Palefsky JM. 2018. E5 can be expressed in anal cancer and leads to epidermal growth factor receptor-induced invasion in a human papillomavirus 16-transformed anal epithelial cell line. J Gen Virol 99:631–644. doi:10.1099/jgv.0.001061. 29624161 PMC6537625

[71] Uberoi A, Yoshida S, Lambert PF. 2018. Development of an in vivo infection model to study mouse papillomavirus-1 (MmuPV1). J Virol Methods 253:11–17. doi:10.1016/j.jviromet.2017.12.002. 29253496 PMC5898621

[72] Darragh TM, Colgan TJ, Cox JT, Heller DS, Henry MR, Luff RD, McCalmont T, Nayar R, Palefsky JM, Stoler MH, Wilkinson EJ, Zaino RJ, Wilbur DC, Members of LAST Project Work Groups. 2012. The lower anogenital squamous terminology standardization project for HPV-associated lesions: background and consensus recommendations from the College of American Pathologists and the American Society for Colposcopy and Cervical Pathology. Arch Pathol Lab Med 136:1266–1297. doi:10.5858/arpa.LGT200570. 22742517

[73] Makielski KR, Lee D, Lorenz LD, Nawandar DM, Chiu Y-F, Kenney SC, Lambert PF. 2016. Human papillomavirus promotes Epstein-Barr virus maintenance and lytic reactivation in immortalized oral keratinocytes. Virology 495:52–62. doi:10.1016/j.virol.2016.05.005. 27179345 PMC4912861

