# Supplementary material for: A Novel Model for Papillomavirus-Mediated Anal Disease and Cancer Using the Mouse Papillomavirus
Source: mBio. 2021 Jul 20;12(4):e01611-21. doi: 10.1128/mBio.01611-21 (PMC8406235; doi:10.1128/mBio.01611-21)
Supplement: FIG S1 [file mbio.01611-21-sf001.pdf]

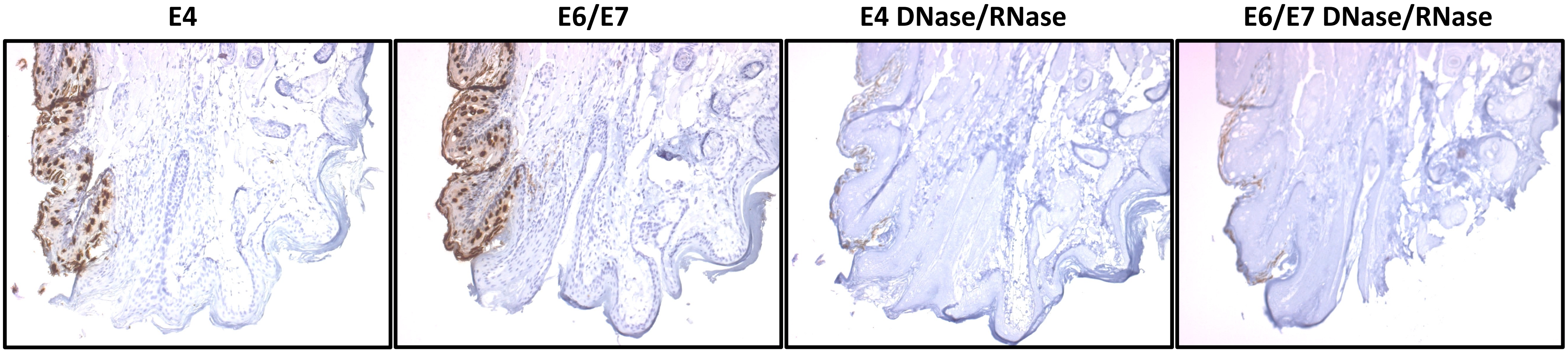

Supplemental Figure 1: Viral signal was not reliably detected within the invasive NSG cancer by E4 or E6/E7 RNAScope. Nuclei were absent for ISH signal, indicating a lack of viral amplification. Trace cytoplasmic signal was detected near the invasive area, but it was not sufficiently above background elsewhere in the tissue to be confidently classified as true signal. All scale bars equal 100 µm.
