# Supplementary material for: A Novel Model for Papillomavirus-Mediated Anal Disease and Cancer Using the Mouse Papillomavirus
Source: mBio. 2021 Jul 20;12(4):e01611-21. doi: 10.1128/mBio.01611-21 (PMC8406235; doi:10.1128/mBio.01611-21)
Supplement: FIG S2 [file mbio.01611-21-sf002.pdf]

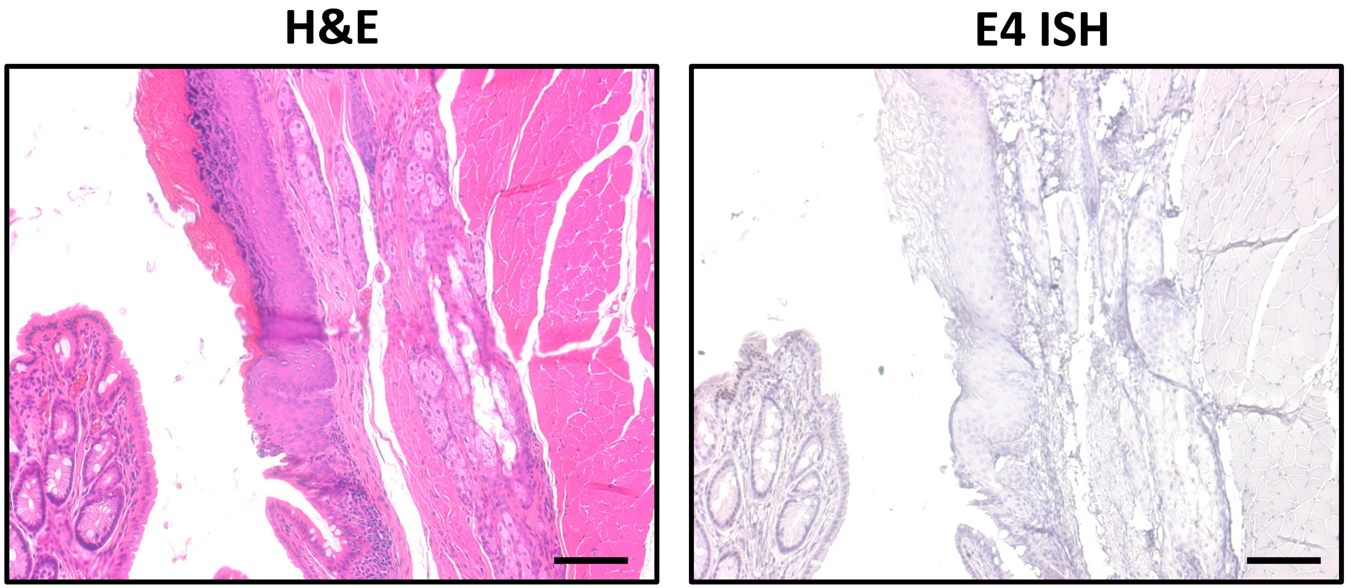

Supplemental Figure 2: A representative lesion showing that viral signal was not definitively detected in LSIL lesions arising in the MmuPV1+UVB group by RNAScope. All scale bars equal 100 µm.
