# Supplementary material for: A Novel Model for Papillomavirus-Mediated Anal Disease and Cancer Using the Mouse Papillomavirus
Source: mBio. 2021 Jul 20;12(4):e01611-21. doi: 10.1128/mBio.01611-21 (PMC8406235; doi:10.1128/mBio.01611-21)
Supplement: FIG S6 [file mbio.01611-21-sf006.pdf]

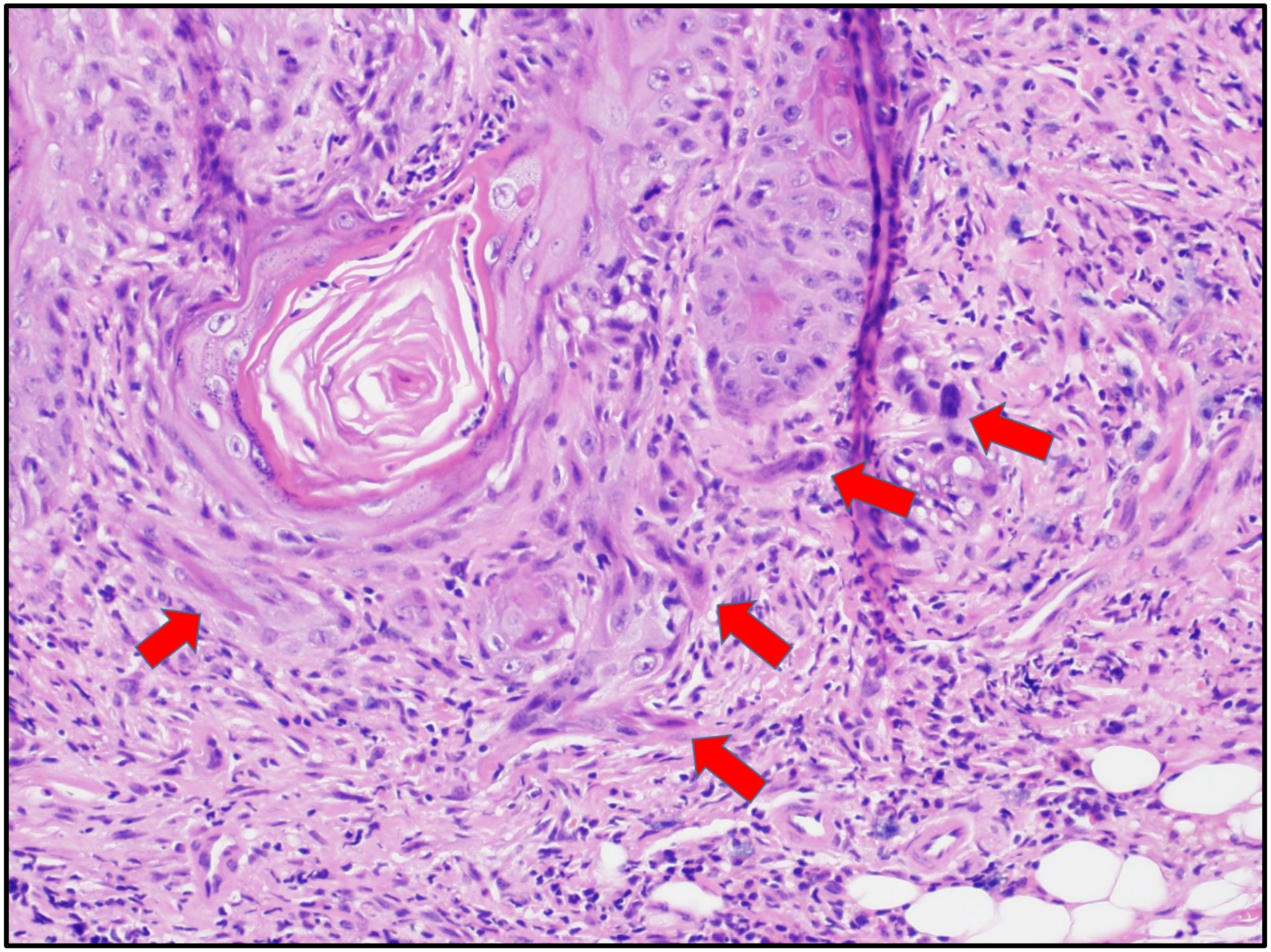

Supplemental Figure 6: Higher magnification image of the cancer shown in Figure 5, with invasive areas indicated with arrows.
