# Supplementary material for: A Novel Model for Papillomavirus-Mediated Anal Disease and Cancer Using the Mouse Papillomavirus
Source: mBio. 2021 Jul 20;12(4):e01611-21. doi: 10.1128/mBio.01611-21 (PMC8406235; doi:10.1128/mBio.01611-21)
Supplement: FIG S3 [file mbio.01611-21-sf003.pdf]

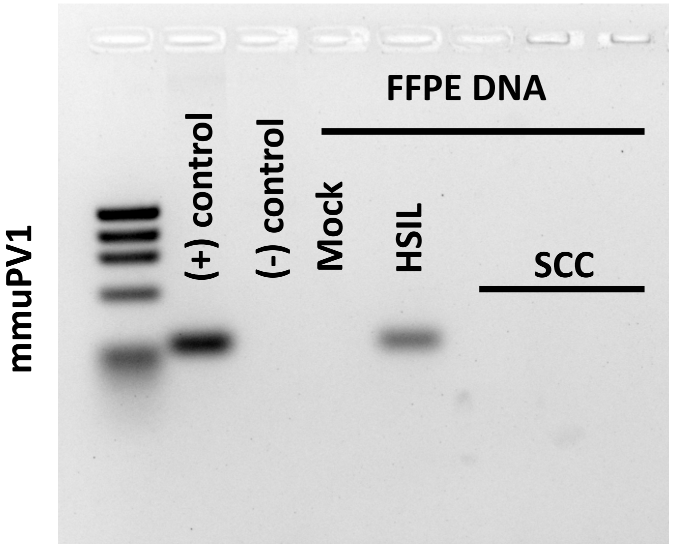

Supplemental Figure 3: FFPE DNA recovered from tissues harboring invasive squamous cell carcinoma was negative for MmuPV1 by PCR. A mock-infected FVB tissue and HSIL lesion from the MmuPV1+DMBA+UVB group were included as negative and positive controls, respectively.
