# Supplementary material for: A Novel Model for Papillomavirus-Mediated Anal Disease and Cancer Using the Mouse Papillomavirus
Source: mBio. 2021 Jul 20;12(4):e01611-21. doi: 10.1128/mBio.01611-21 (PMC8406235; doi:10.1128/mBio.01611-21)
Supplement: FIG S4 [file mbio.01611-21-sf004.pdf]

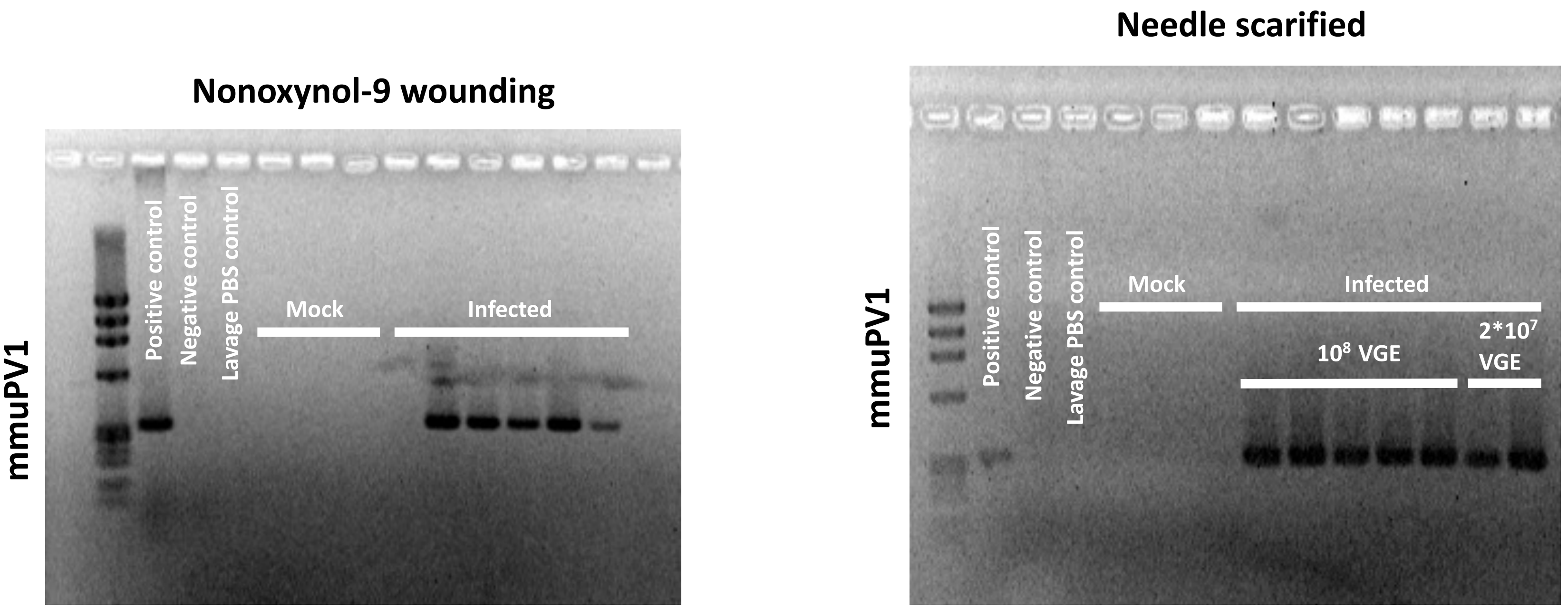

Supplemental Figure 4: Lavages of mice chemically wounded with nonyxonol-9 or physically wounded by needle scarification were analyzed for MmuPV1 by PCR, showing that these alternative anal infection methods also efficiently infected NSG mice.
