# Supplementary material for: A Novel Model for Papillomavirus-Mediated Anal Disease and Cancer Using the Mouse Papillomavirus
Source: mBio. 2021 Jul 20;12(4):e01611-21. doi: 10.1128/mBio.01611-21 (PMC8406235; doi:10.1128/mBio.01611-21)
Supplement: FIG S5 [file mbio.01611-21-sf005.pdf]

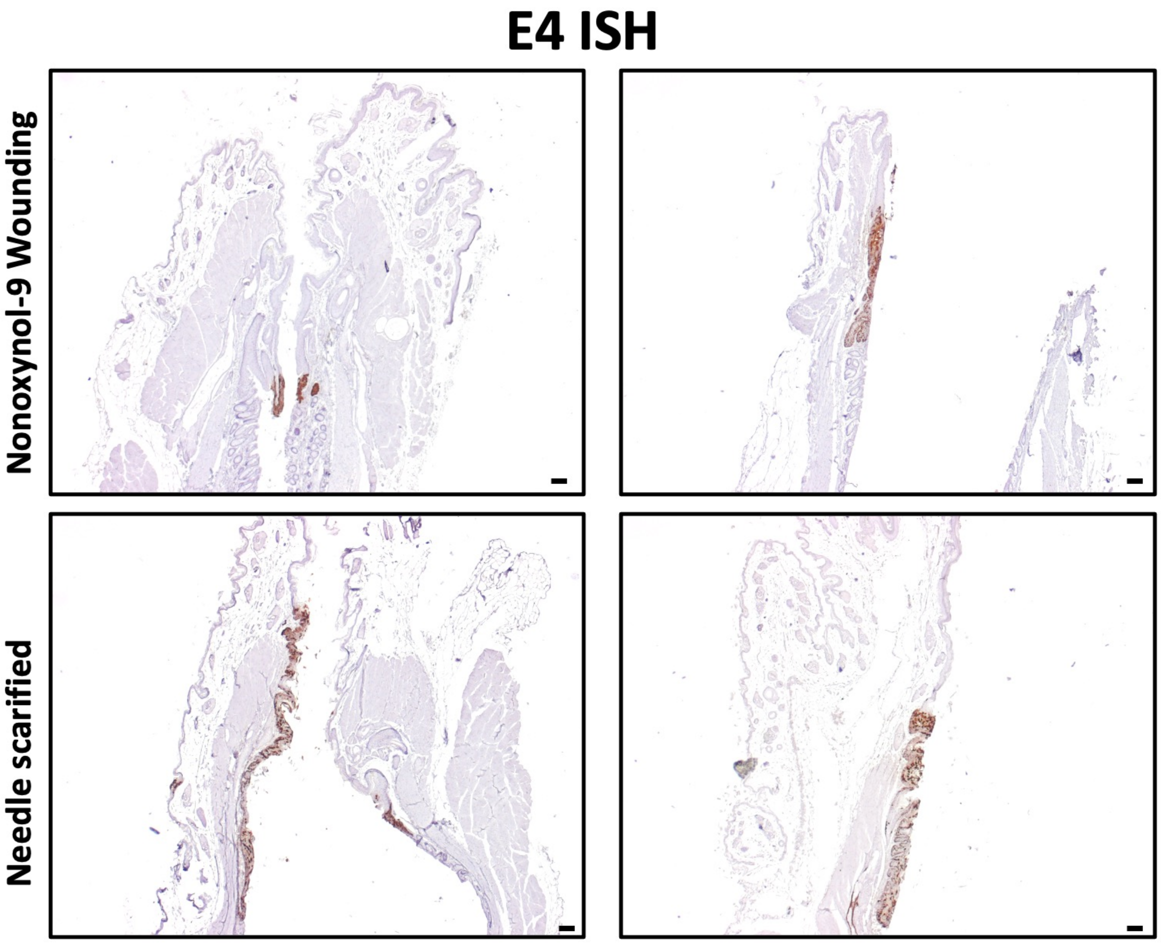

Supplemental Figure 5: Representative tissues showing the full areas of viral infection in alternatively wounded NSG mice at the 3 months study endpoint by E4 RNAScope ISH. All scale bars equal 100 µm.
